# Comparative Bioremediation of Tetradecane, Cyclohexanone and Cyclohexane by Filamentous Fungi from Polluted Habitats in Kazakhstan

**DOI:** 10.3390/jof10060436

**Published:** 2024-06-19

**Authors:** Mariam Gaid, Wiebke Jentzsch, Hannah Beermann, Anne Reinhard, Mareike Meister, Ramza Berzhanova, Togzhan Mukasheva, Tim Urich, Annett Mikolasch

**Affiliations:** 1 Institute of Microbiology, University Greifswald, Felix-Hausdorff-Straße 8, 17489 Greifswald, Germany; 2 Leibniz Institute for Plasma Science and Technology (INP), Felix-Hausdorff-Str. 2, 17489 Greifswald, Germany; 3 Department of Biology and Biotechnology, Al-Farabi Kazakh National University, Al-Farabi Ave 71, Almaty 050040, Kazakhstan

**Keywords:** alkanes, crude oil, cyclohexane, cyclohexanone, diterminal oxidation, mycoremediation

## Abstract

Studying the fates of oil components and their interactions with ecological systems is essential for developing comprehensive management strategies and enhancing restoration following oil spill incidents. The potential expansion of Kazakhstan’s role in the global oil market necessitates the existence of land-specific studies that contribute to the field of bioremediation. In this study, a set of experiments was designed to assess the growth and biodegradation capacities of eight fungal strains sourced from Kazakhstan soil when exposed to the hydrocarbon substrates from which they were initially isolated. The strains were identified as *Aspergillus* sp. SBUG-M1743, *Penicillium javanicum* SBUG-M1744, SBUG-M1770, *Trichoderma harzianum* SBUG-M1750 and *Fusarium oxysporum* SBUG-1746, SBUG-M1748, SBUG-M1768 and SBUG-M1769 using the internal transcribed spacer (ITS) region. Furthermore, microscopic and macroscopic evaluations agreed with the sequence-based identification. *Aspergillus* sp. SBUG-M1743 and *P. javanicum* SBUG-M1744 displayed remarkable biodegradation capabilities in the presence of tetradecane with up to a 9-fold biomass increase in the static cultures. *T. harzianum* SBUG-M1750 exhibited poor growth, which was a consequence of its low efficiency of tetradecane degradation. Monocarboxylic acids were the main degradation products by SBUG-M1743, SBUG-M1744, SBUG-M1750, and SBUG-M1770 indicating the monoterminal degradation pathway through β-oxidation, while the additional detection of dicarboxylic acid in SBUG-M1768 and SBUG-M1769 cultures was indicative of the fungus’ ability to undertake both monoterminal and diterminal degradation pathways. *F. oxysporum* SBUG-M1746 and SBUG-M1748 in the presence of cyclohexanone showed a doubling of the biomass with the ability to degrade the substrate almost completely in shake cultures. *F. oxysporum* SBUG-M1746 was also able to degrade cyclohexane completely and excreted all possible metabolites of the degradation pathway. Understanding the degradation potential of these fungal isolates to different hydrocarbon substrates will help in developing effective bioremediation strategies tailored to local conditions.

## 1. Introduction

Kazakhstan, with its significant energy reserves, has emerged as a pivotal partner in fulfilling the energy security of the EU [1,2]. Globally, crude oil consumption remains paramount (>31%; [3]) among other energy sources such as natural gas, coal, nuclear energy, hydroelectricity and renewable sources. The pollution of ecosystems by crude oil and its products is an expected outcome of the exponential growth in industrial activities over the last century [4,5]. Due in part to inefficient or flawed extraction methods, transportation accidents, and processing errors, hydrocarbon pollutants have found their way into various environmental matrices [4]. Recently, it has been reported that a daily oil output of 61,165 barrels makes 3% oil spill in the Gulf of Mexico [6]. Such frequent events highlight the urgent need for effective strategies accompanied with robust industrial protocols that adopt advancements in renewable energy to curtail the dependency on crude oil. This may alleviate the damage to the environment and wildlife.

Alkanes, cycloalkanes and aromatic hydrocarbons constitute the majority of the complex organic mixture of crude oil [7]. The natural breakdown of these residual hydrocarbons mainly relies on earth’s microorganisms that orchestrate the biodegradation processes. Bioremediation is an environmentally sustainable and cost-effective tool where microorganisms, either naturally endemic to polluted areas or intentionally introduced, are integrated to diminish environmental contaminants [8,9,10].

While several contemporary studies have focused on the bioremediation capabilities of bacterial strains, the potential contributions of fungi are increasingly recognized [11,12,13]. Emerging terms like “mycoremediation” and “mycodegradation” highlight the specificity of fungal-based degradation techniques in treating organic pollutants. These processes leverage both the intra- and extracellular enzyme systems and organic acids produced by fungi [14,15,16,17,18,19,20,21].

Oil reserves in Kazakhstan are estimated to support extraction and production for many decades [22]. The sites where oil is extracted often suffer from contamination. Thus, understanding microbial degradation mechanisms can open avenues to reduce the impact of crude oil residues on marine or terrestrial habitats [23,24].

On this background, we had isolated highly potent bacterial oil degraders from contaminated soils of deposits in Kazakhstan. These isolates utilized a large number of oil components and by degrading pollutants they had beneficial effects on the growth of barley seeds in the presence of crude oil [25,26]. Now the question has been raised as to whether filamentous fungi of the same biodegradation potential can be isolated from deposits or polluted areas in Kazakhstan. For this reason, different filamentous fungi should be isolated on a mineral medium containing either typical hydrocarbons such as the well utilizable aliphatic *n*-alkane tetradecane, the oxygenated derivative of alicyclic hydrocarbons cyclohexanone, or crude oil itself. Many microorganisms can oxidize cyclohexane to cyclohexanone, but further metabolism of the oxygenated alicyclic cyclohexanone is difficult for many organisms [27]. An additional aim of this study was therefore to identify effective fungal degraders for cyclohexanone, which is otherwise difficult to degrade. In this context, the possibly isolated cyclohexanone degraders should then also be tested to see whether they could degrade the non-oxidized parent compound cyclohexane in order to find likely fungal strains that are able to completely degrade cycloalkanes and use them as the sole source of carbon and energy.

Therefore, the current study delves into the exploration of fungal strains belonging to four genera and degrading oil components. The identified strains originate from diverse locales within Kazakhstan, including regions of active oil production. These strains were analyzed to determine whether they can biodegrade their isolation substrates in vitro. The substrates exemplified the model components of crude oil; tetradecane as a model for *n*-alkanes and cyclohexanones for alicyclic hydrocarbons.

## 2. Materials and Methods

### 2.1. Preparation of Fungal Cultures and Their Identification

**Figure 1:**
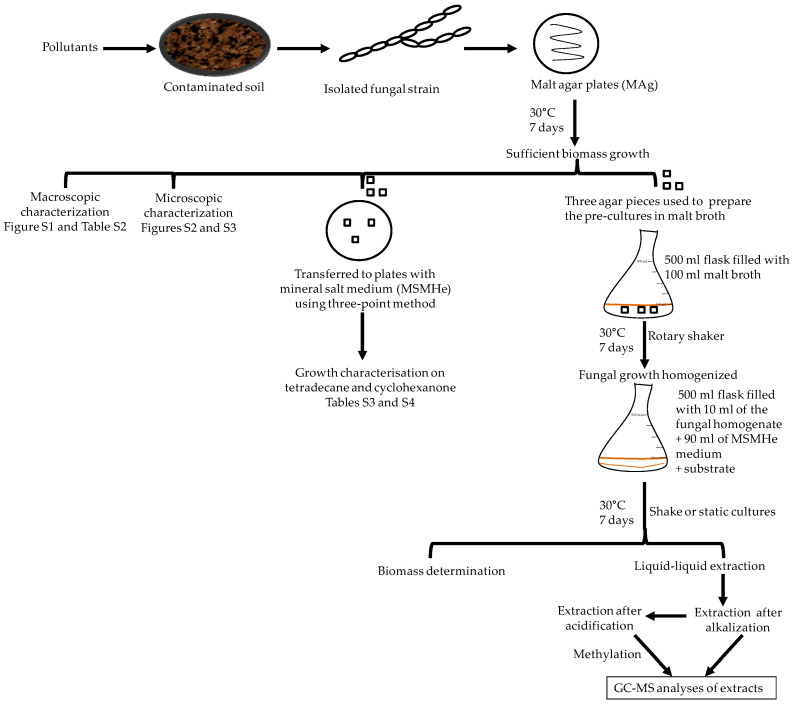
An overview of the methodology and approaches used in the current study.

Eight filamentous fungal strains were isolated from various soil samples in Kazakhstan. The fungi were enriched on the substrates tetradecane, cyclohexanone, and crude oil, each with mineral salt medium (MSMHe) according to a previously described method [28]. They belong to the strain collection of the Department of Biology at the University of Greifswald at the Institute for Microbiology (SBUG). Strains SBUG-M1743, SBUG-M1748 and SBUG-M1750 were isolated from a park near the Almaty train station (43°20′36.8″ N 76°56′30.7″ E). Oil depot “Ozen” was the source of SBUG-M1746 (43°26′36″ N 52°46′56″ E), while Aktöbe oil deposit (50°20′38.2″ N 57°05′11.0″ E) presented the source of SBUG-M1744, SBUG-M1768, SBUG-M1769, and SBUG-M1770. The isolation substrate of SBUG-M1743, SBUG-M1750 and SBUG-M1744 was tetradecane, while cyclohexanone was identified as the isolation substrate of SBUG-M1746 and SBUG-M1748, and crude oil for SBUG-M1768, SBUG-M1769 and SBUG-M1770. MSMHe and malt agar (MAg) plates were used to cultivate the fungal cultures as previously described by Gaid et al. [12]. The fungi were inoculated on MAg in petri dishes using the three-point method and then incubated for seven days at 30 °C. With the three-point method, three approximately 1 × 1 cm large pieces of agar grown with the fungus were sterilely cut from a well-grown MAg plate and distributed evenly on a new plate. A 7-day-old plate incubated with cell material was fixed as a starting plate for all further procedures. Microscopic features of each strain were examined using Leica DM 2500 LED microscope (Wetzlar, Germany) as described previously [12], while phenotyping via macroscopic examination of the cultures grown on MAg plates was used to determine their filamentous nature. A simplified overview of the culturing methodology is depicted in Figure 1. The extraction and purification of fungal DNA for the molecular identification of the strains were performed following the manufacturer instructions (DNeasy^®^ PowerSoil^®^ Kit (Qiagen, Hilden, Germany). Gene sequences of internal transcribed spacer (ITS) were identified following the steps described previously [12]. The obtained sequence data were examined via NCBI-ITS (https://www.ncbi.nlm.nih.gov/, accessed on 20 February 2024).

### 2.2. Growth Experiment on the Fungal Isolation Substrates

The growth experiments of the strains took place with respect to the substrates tetradecane or cyclohexanone (Sigma-Aldrich, Taufkirchen, Germany). SBUG-M1743, SBUG-M1744, SBUG-M1750, SBUG-M1768, SBUG-M1769 and SBUG-M1770 strains were examined for their growth on tetradecane as a carbon and energy source. Five MSMHe glass plates were prepared for each strain. Whatman™ cellulose filter paper (110 mm diameter; Cytiva, Bronshoj, Denmark) was introduced into the lid of each plate. One ml of tetradecane was pipetted onto the filter paper of each plate. The filter of the control plate remained untreated. Plates that were loaded with the substrate were incubated together at 30 °C over 7 days in a glass container to saturate its air with the substrate. Additional 0.5 mL of tetradecane was added to each filter paper on the fifth day, while control plates remained untreated and were incubated in a separate, but similar container under same conditions. Cultures of SBUG-M1746 and SBUG-M1748 cultivated in MSMHe glass plates were tested for their ability to use and grow on cyclohexanone as a carbon and energy source. This substrate is a volatile liquid and was rapidly transitioned to the gas phase at 30 °C. Thus, no filter papers were introduced into the lids of the petri dishes. Instead, the inoculated plates were exposed to the vapor of 2 mL of cyclohexanone placed in an open beaker. The plates were incubated at 30 °C for 7 days in one glass container with the cyclohexanone beaker. This container was tightly sealed with Parafilm^®^ (Bemis Company, Neenah, WI, USA). Control plates were incubated in a separate glass container without substrate under same conditions. For both substrates, the assessment of strain growth took place after 5 and 7 days using three-point and streak methods for all plates.

### 2.3. Biodegradation

The cultivation of the eight fungal strains for biodegradation experiments and corresponding control cultures was performed in malt broth followed the method described by Gaid et al. [12]. For incubation with tetradecane, eight sterile 500 mL wide-neck round-bottom flasks with cotton plugs were used. Conversely, for the attempts with cyclohexanone and cyclohexane (Sigma-Aldrich, Taufkirchen, Germany), narrow-neck round-bottom flasks with ground-glass stoppers were required due to the high volatility of the substrates. In all three incubation approaches, six flasks were filled with 90 mL of MSMHe, serving as biodegradation and cell controls (without substrate). In two flasks per approach, 100 mL of MSMHe was filled without cell material (substrate controls). In addition, 1 mL of the vitamin stock solution was included in all flasks. Tetradecane was used at concentration of 0.25 mL/100 mL for the initial screening experiments followed by 0.5 mL/100 mL medium for the main experiments. However, due to the highly volatile nature of cyclohexanone and cyclohexane, the substrates were added just before incubation in a concentration of 0.25 mL/100 mL medium. Substrate was pipetted into six flasks (biodegradation approaches and substrate controls) after sterile filtration (Minisart SRP 25, pore size 0.2 μm, Sartorius Stedim Biotech GmbH, Göttingen, Germany) 1 h before incubation began. The flasks were swirled to ensure the distribution of the substrate. After 1 h, four flasks with substrate (biodegradation approaches) and two without substrate (cell controls) were inoculated with 10 mL of cell homogenate of the malt broth pre-cultivation using a pipette tip cut at the end. Two flasks from the total of eight, to which only substrate and no cells were added, served as substrate controls. All approaches were incubated for 7 days at 30 °C. Four flasks (one flask with cell homogenate but without substrate, one flask without cell homogenate but with substrate, two flasks with cell homogenate and substrate) were shaken at 130 rpm in a rotary shaker (shaking cultures). Similarly, another four flasks were incubated as stationary cultures for the same period and at the same temperature (static cultures) as exemplified in Figure 1.

### 2.4. Determination of the Biomass

The biomass was measured as dry weight as described previously [12]. Cell suspension was filtered under vacuum through an oven-dried and weight-determined filter. For the determination of the cell weight of the biodegradation experiments (cultures with substrate and cultures without substrate = cell control), all biodegradation samples (100 mL) were filtered. For the determination of the cell weight of the start biomass, 5 mL of the homogenate of the pre-culture were filtered. The filters were then dried for 24 h at approximately 100 °C, weighed with the precision balance (OHAUS Europe, Greifense, Switzerland), and the weight difference between the filter weights with biomass and the weight of an empty filter yielded the weight of the biomass. The cell weight of the start biomass was doubled because 10 mL of homogenate was used.

### 2.5. Extraction and Identification of the Biodegradation Products

After seven days of incubation, substrate degradation by filamentous fungal strains were investigated in the cell-free supernatants (except for substrate controls that were used directly as they devoid of cell material).

Following the method described by Mikolasch et al. [26], the degradation products were isolated using liquid–liquid extraction to prepare extracts after alkaline and acidic extraction. The obtained extracts after acidic extraction were methylated prior to their analysis via gas chromatography–mass spectrometry (GC-MS, Figure 1).

## 3. Results

### 3.1. Identification of the Fungal Strains

The diversity of the isolated fungal strains was confirmed not only for their ability to degrade the isolation substrate in vitro, but also for their taxonomical ranking. For this purpose, their ITS sequences were surfed against publicly available NCBI databases using the Basic Local Alignment Search Tool (BLAST; Table S1). The SBUG-M1743 (St1) strain was isolated on tetradecane and identified as *Aspergillus* sp. Since various species appeared in the BLAST results, this strain was named up to the genus level. Two more strains were isolated on tetradecane as substrate, SBUG-M1744 (St2) and SBUG-M1750 (St3) and identified as *Penicillium javanicum* and *Trichoderma harzianum,* respectively. The strains that were isolated on cyclohexanone, SBUG-M1746 (St4) and SBUG-M1748 (St5), belong to the *Fusarium* genus. Blast results showed that both strains are *Fusarium oxysporum*. Three strains isolated on crude oil, SBUG-M1768 (St6) and SBUG-M1769 (St7) were identified as *Fusarium oxysporum,* and SBUG-M1770 (St8) as *Penicillium javanicum* (Table 1). The accession numbers of ITS sequences of the newly identified strains are available at NCBI: *Aspergillus* sp. St1 (OR335321), *F. oxysporum* St4 (PP227933), *F. oxysporum* St5 (PP227942), *F. oxysporum* St6 (PP227940), *F. oxysporum* St7 (PP227939), *P. javanicum* St2 (PP227941), *P. javanicum* St8 (PP227938), and *T. harzianum* St3 (OR335320).

In addition to the use of molecular markers to identify the strains, visual assessment helped to identify the typical growth and habitus of the various filamentous fungal strains (Table S2). Despite sharing the same isolation substrate, tetradecane, *Aspergillus* sp. St1 and *T. harzianum* St3 showed clear distinguishing characteristics in color, diameter, and morphology (Figure S1a,c). On the other hand, *P. javanicum* St2 grew similarly to its congeners St8 (Figure S1b,h). Comparably, the four *F. oxysporum* strains isolated on cyclohexanone (St4, and St5; Figure S1d,e), and crude oil (St6, and St7; Figure S1f,g) were almost identical, supporting the sequencing results that how these strains belong to the same species. All strains showed visible to strong growth on the MAg plates (Table S2). On the other hand, moderate growth was observed in the malt broth pre-cultures of the four *Fusarium* strains St4, St5, St6 and St7, while limited growth was a characteristic feature of *T. harzianum* St3. Comparable to their growth on MAg plates, the pre-cultures of *Aspergillus* sp. St1 and *P. javanicum* St2, St8 showed strong growth.

Additional to macroscopic examination of the fungal strains, microscopic evaluation also agreed with the molecular identification. After 3 or 7 days of growth, *Aspergillus* sp. St1 showed hyphae (conidiophores) terminating into swollen round vesicles. Metulae and phialides were at the surface of these vesicles, from which smooth round conidiospores emerged and spread on the entire 7-day-old plate. The entire arrangement of conidiophore-hyphal structures constituted the mycelial network (Figure S2a). *P. javanicum* St2 formed hyphae with attached phialides after 3 days. The shaped form of phialides was notable, from which smooth round conidiospores also emerged. After 7 days, the formation of large cleistothecia, perceivable with the naked eye as grains of sand, was notable. Crushing cleistothecia released the internal asci after exposing the elliptical spiky ascospores (Figure S2b). Microscopical evaluation of *T. harzianum* St3 revealed the formation of long hyphae with phialides arranged like conidiophores with a (complicated) dendroid branching system. At the ends of the phialides, conidia subglobose to short oval shape could be detected (Figure S2c). Slender conidiophores and attached phialides were the distinct features of *F. oxysporum* St4 after both 3 and 7 days of growth. Compared to the previous strains, these conidiospores had an elongated oval shape. After 7 days, the formation of countless new conidiospores was apparent and the beginning of cell red pigmentation was observed (Figure S2d). The cell development and morphology of *F. oxysporum* St5 closely resembled those of *F. oxysporum* St4. After 3 days of growth, the slender conidiophore and attached phialides, as well as countless elongate conidiospores, were visible. After 7 days, the elongated form of the conidiospores was notably enlarged, which resembled the shape of beans (Figure S2e). After a culture period of seven days, *F. oxysporum* St6 and St7 presented numerous oval to slightly teardrop-shaped microconidia, along with some y septate curved macroconidia (Figure S3a,b). The hyphae exhibited bottle-shaped phialides of various sizes. Additionally, intercalary chlamydospores were detected in micrographs of *F. oxysporum* St7 (Figure S3c,d). The ochre-colored fruiting bodies were recognized in plates harboring *P. javanicum* St8 strain after 7 days of growth. They contain oval, hyaline asci with eight oval ascospores each (Figure S3e,f).

### 3.2. Growth of the Fungal Strains on Hydrocarbons

These experiments aimed to investigate the growth of filamentous fungal strains with tetradecane (Table S3) and cyclohexanone (Table S4). A control sample without any carbon source was used for reference. St1, St2, St3, St4, and St5 were grown on their isolation carbon sources. The other three strains St6, St7, and St8 were isolated on crude oil, but the growth on agar plates was performed using tetradecane as model substrate for *n*-alkanes, which is one of the main components constituting the crude oil. The filamentous fungi *Aspergillus* sp. St1, *P. javanicum* St2, St8 and *F. oxysporum* St6, St7 showed visible growth after 5 days, which increased further or remained unchanged after 7 days of incubation. It was therefore hypothesized that these strains can utilize tetradecane as the sole carbon source and form metabolites. A possible poor utilizer of tetradecane was the filamentous fungus *T. harzianum* St3, which was evidenced by the no and weak growth after 5 and 7 days, respectively (Table S3). Thus, it was presumed that this strain might form fewer or no biodegradation products of tetradecane compared to *Aspergillus* sp. St1 and *P. javanicum* St2. The two filamentous fungi, *F. oxysporum* St4 and St5, exhibited visible growth after 7 days in the presence of cyclohexanone as the sole carbon source (Table S4). It was therefore speculated that these two fungal strains might be able to degrade cyclohexanone. Control plates showed very weak to no growth.

### 3.3. Ability of the Fungal Strains to Biodegrade Hydrocarbons

The utilization of the *n*-alkane tetradecane or the cyclic ketone cyclohexanone as carbon and energy sources influenced the cell growth of the strains, which was evaluated by measuring the formed biomass. The consumption of substrates by the tested strains lead to formation of new metabolites or biodegradation products, which were verified by GC-MS analyses of the cell-free extracts. No degradation products could be detected in extracts prepared at pH 9 from tetradecane-containing cultures, while extracts prepared at pH 2 showed significant differences in the pattern of biodegradation products when compared to substrate controls (i.e., control without cells). On the other hand, the detection of biodegradation products in both extracts prepared from cultures growing on cyclohexanone was possible. Biomass change and product formation after growing on tetradecane were investigated for *Aspergillus* sp. St1, *P. javanicum* St2, St8, *T. harzianum* St3, and *F. oxysporum* St6, St7, while *F. oxysporum* St4 and St5 were tested after their growth on cyclohexanone.

#### 3.3.1. Biodegradation of Tetradecane

A screening with 0.25% tetradecane for the ability to degrade this substrate in liquid medium as single source of carbon and energy was first carried out for the six-tetradecane degraders (Figure S4). All strains were able to degrade tetradecane. Although the degradation rates varied from strain to strain, the remaining substrate was less than 40%.

**Figure 2:**
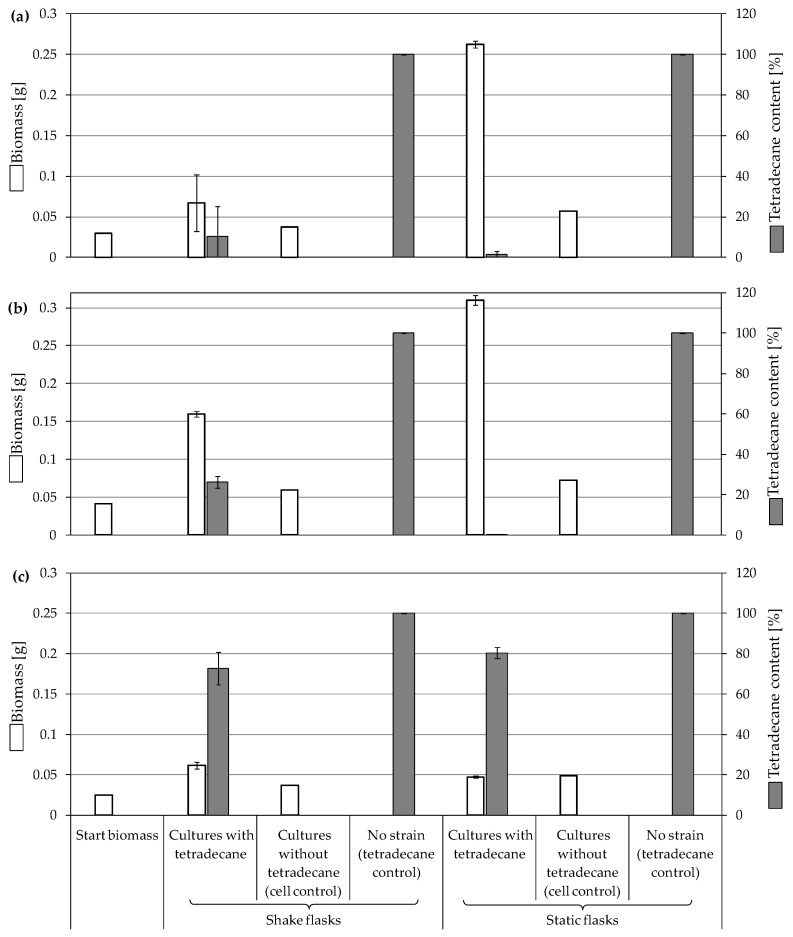
Growth of fungal strains on 0.5% tetradecane and the remaining substrate after 7 days of incubation. (**a**) *Aspergillus* sp. St1, (**b**) *P. javanicum* St2, and (**c**) *T. harzianum* St3. Residual growth in cell controls was due to the pre-cultivation in malt broth. Bars presenting the mean values ± SD (n = 2).

The representative examples *Aspergillus* sp. St1, *P. javanicum* St2 and *T. harzianum* St3 were further explored for the growth changes and biodegradation ability of this substrate (0.5%; Figure 2). These three strains were selected as exemplary samples for the best and worst tetradecane consumers.

**Figure 3:**
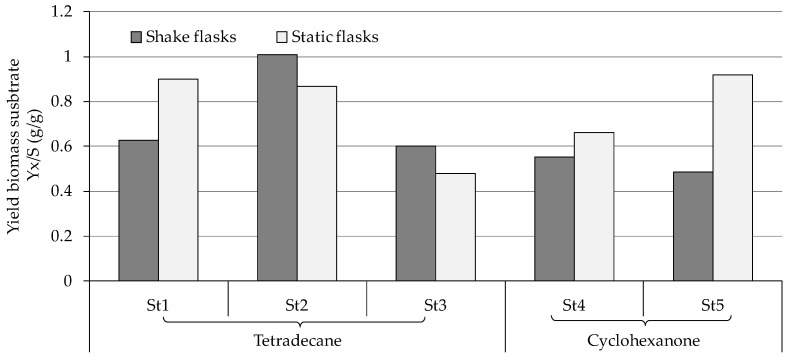
Yield biomass-substrate (Yx/S) of *Aspergillus* sp. St1, *P. javanicum* St2, *T. harzianum* St3, *F. oxysporum* St4, and *F. oxysporum* St5 after incubation with tetradecane or cyclohexanone. Data are the division results from values presented in Figure 2, Figure 4 and Figure 5.

*Aspergillus* sp. St1 showed pronounced degradation of the substrate both at 0.25% and at 0.5% tetradecane (Figure 2 and Figure S4). In both shake and static cultures, the remaining substrate did not exceed 11% (Figure 2a). Different from the shake cultures biomass that increased by double, the biodegradation approaches of the static cultures showed a 9-fold biomass increase from the starting biomass combined with the almost complete degradation of the substrate (Figure 2a). This caused the yield biomass-substrate of the static cultures to be 30% higher than its shake counterpart (Figure 3). The better biomass production and substrate use in standing cultures could also be confirmed visually on the basis of the compact mycelium layer at the medium surface compared to the corresponding layer in shaking cultures and therefore more diffuse cell material in the whole shaking cultures (Figure S5a,b). Cell controls of shake and static cultures showed no or weak increase in the biomass, respectively, without any mycelium layer at the medium surfaces. The residual growth in the cell controls was due to pre-cultivation in malt broth.

*P. javanicum* St2 exhibited complete tetradecane degradation at 0.25% tetradecane and in the static culture of 0.5% tetradecane (Figure 2b and Figure S4). The exception was the shaking cultures with 0.5% tetradecane, as approximately 26% of remained substrate was measured. The biodegradation approach of the static cultures showed a strong biomass increase with an average 7.5-fold increment from the starting biomass (Figure 2b). Shaking culture biodegradation exhibited almost 4-fold biomass increase resulting in comparable yield biomass-substrate of both type of cultures (Figure 3). The controls without substrate achieved indistinguishable biomass growth. For this strain, too, the better biomass production in standing cultures could be visually verified on the basis of compact mycelium layer at the medium surface compared to less and diffuse cell material in shaking cultures (Figure S5c,d).

Due to the poor growth of *T. harzianum* St3 on tetradecane (Section 3.2, Table S3), the strain was ranked as the poorest utilizer among the tested strains on the same isolation substrate (Figure 2c and Figure S4). Accordingly, biomass growth was minimal in all samples of this strain, which could already be deduced visually from the absence of mycelium layers and the only slightly diffuse cell material (Figure S5e). Around 36% remaining substrate was detected after incubating the strain with 0.25% tetradecane (Figure S4), while 72.7% and 80% remaining substrate were measured after shake and static incubation with 0.5% tetradecane, respectively (Figure 2c). Similarly, the yield biomass-substrate of St3 was the lowest compared to the other strains tested on the same substrate (Figure 3).

The two *F. oxysporum* isolates St6 and St7 exhibited comparable substrate consumption (<20% remained substrate; Figure S4). Similar to *P. javanicum* St2, *P. javanicum* St8 showed complete substrate utilization at 0.25% tetradecane (Figure S4). In biodegradation experiments of *Aspergillus* sp. St1, the formation of five monocarboxylic acids (i.e., tetradecanoic, dodecanoic, decanoic, octanoic and hexanoic acids) was apparent in both incubation methods as products of the monoterminal degradation pathway of tetradecane (Table 2). *P. javanicum* St2 could degrade tetradecane to tetradecanoic, dodecanoic, and octanoic acid in both static and shake biodegradation approaches. Tetradecanoic, and dodecanoic acids were the degradation products of *T. harzianum* St3 in both culturing approaches (Table 2). Additionally, decanoic and octanoic acids could be detected in extracts prepared from the static cultures, while hexanoic acid was exclusively present in shake cultures (Table 2).

In the degradation of tetradecane by *F. oxysporum* St6 and St7, five metabolites were detected. These acids were dodecanoic, decanoic, octanoic, and hexanoic acid, which may indicate the monoterminal degradation pathway and the dicarboxylic acid hexanedioic acid, which may indicate the diterminal degradation pathway (Table 3). Despite the ability of *P. javanicum* St8 to completely utilize tetradecane (Figure S4), only three monocarboxylic acids were detected as products (Table 3).

#### 3.3.2. Biodegradation of Cyclohexanone

**Figure 4:**
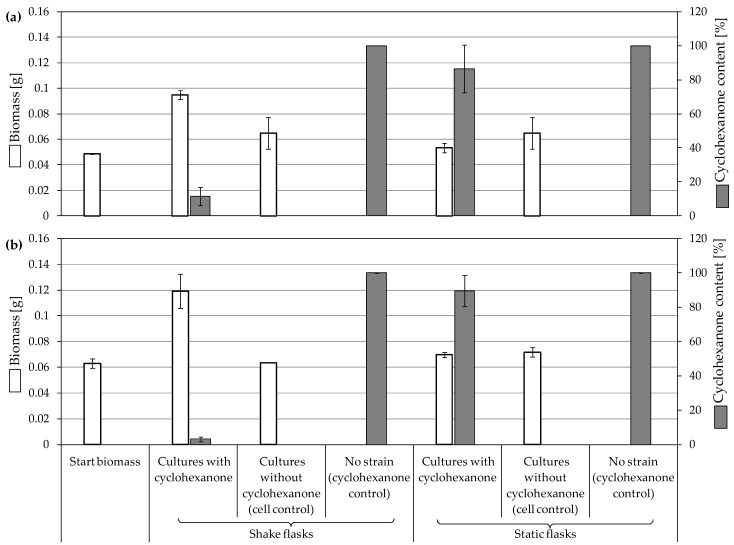
Growth of fungal strains on 0.25% cyclohexanone and the remaining substrate after 7 days of incubation. (**a**) *F. oxysporum* St4, and (**b**) *F. oxysporum* St5. Residual growth in cell controls was due to the pre-cultivation in malt broth. Bars presenting the mean values ± SD (n = 2).

The two strains incubated with cyclohexanone exhibited a very similar behavior in terms of substrate degradation and biomass formation, albeit with double the yield of biomass-substrate in static cultures of St5 (Figure 3). *F. oxysporum* St4 showed pronounced substrate degradation in the shaking cultures with around 11.6% remaining substrate (Figure 4a). However, the static cultures experienced little substrate degradation (86.5% remained substrate). Biomass growth was moderate in shaking biodegradation showing an approximate doubling of the starting biomasses and was weak in static cultures (Figure 3 and Figure 4a), which agreed with the visible turbid cell suspension in shaking cultures (Figure S6a,b).

*F. oxysporum* St5 showed almost complete degradation of the substrate in the shaking cultures (3% remained substrate; Figure 4b). However, the static cultures exhibited remarkably lower substrate degradation (89.5% remained substrate) and a lower biomass increase than in the cell control. Biomass growth in shaking biodegradation showed an approximate doubling of the starting biomasses, which was also confirmed by considerably more turbid cell suspensions compared to the static cultures (Figure S6c,d). The cell controls of both biodegradation approaches showed no growth.

During the evaluation of the GC-MS profiles, products of cyclohexanone were found in all extracts prepared from *F. oxysporum* St4 and St5 cultures. ε-Caprolactone, belonging to the group of cyclic esters, and cyclohexanol, belonging to the group of secondary alcohols, were detected after alkaline extraction. Additionally, 6-hydroxyhexanoic acid (the free acid of ε-caprolactone) from shake cultures approach and hexanedioic acid (a dicarboxylic acid) from both shake and static cultures were the degradation products that were detected in the acidic extracts (Table 4).

#### 3.3.3. Biodegradation of Cyclohexane

**Figure 5:**
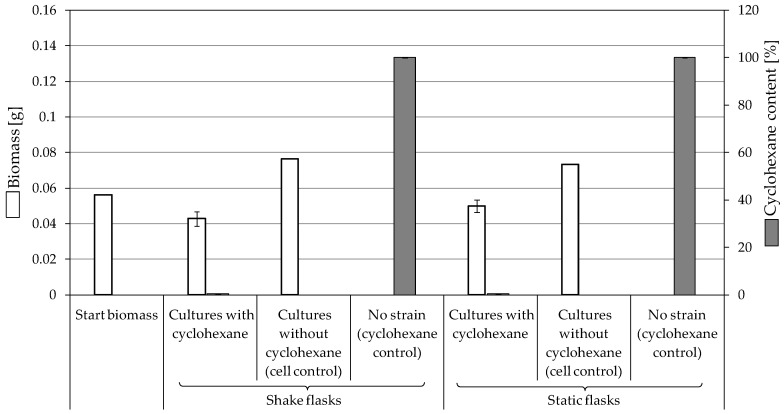
Growth of fungal strains on 0.25% cyclohexane and the remaining substrate after 7 days of incubation with *F. oxysporum* St4. Residual growth in cell controls was due to the pre-cultivation in malt broth. Bars presenting the mean values ± SD (n = 2).

*F. oxysporum* St4 was also able to degrade almost all cyclohexane in shake and static cell cultures (Figure 5). Despite the ability of the fungal strain to consume the whole amount of the substrate, an observed decrease by 24% and 11% of the starting biomass in shake and static cell cultures, respectively, suggested a toxic effect of the substrate. Different from the relatively high yield of biomass-substrate of other strains (Figure 3), the shift of the yield to negative scale (−0.32 for shake cultures and −0.125 g/g for static cultures) further evidences that cyclohexane affects the viable growth of St4.

The ability of *F. oxysporum* St4 to degrade cyclohexane was confirmed by the detection of the same degradation products after the incubation with cyclohexanone in addition to cyclohexanone itself (Table 5). All biodegradation products were absent in cell-free substrate control cultures.

## 4. Discussion

From three samples of different contaminated soils, we isolated eight filamentous fungal strains and described them as oil component degraders. These strains belong to the fungal divisions *Ascomycota* (*F. oxysporum* St4, St5, St6, St7, *P. javanicum* St2, St8, *T. harzianum* St3) and *Fungi imperfecti* (*Aspergillus* sp. St1). The behavior of six strains (*Aspergillus* sp. St1, *P. javanicum* St2, St8, *T. harzianum* St3, and *F. oxysporum* St6, St7) was examined in the presence of tetradecane, while two strains (*F. oxysporum* St4, St5) were examined on cyclohexanone with an additional study of *F. oxysporum* St4 on cyclohexane.

### 4.1. Consumption and Degradation of Tetradecane

The six strains *Aspergillus* sp. St1, *P. javanicum* St2, St8, *T. harzianum* St3, and *F. oxysporum* St6, St7 were grown in the presence of tetradecane. When biomass formation was compared to substrate degradation rate, a clear pattern emerged; the more substrate that could be degraded, the higher the increase in biomass production was. The biomass increase was higher in static cultures than shake cultures. This might be explained by the formed mycelium layer on the surface covering the liquid medium that might help in preventing tetradecane from evaporation and offering the fungi better accessibility to the carbon and energy sources. Furthermore, the shear forces during shaking reduce mycelium formation in the entire flask, which results also in lower biomass production. Except for *T. harzianum* St3 that showed poor increase in the biomass, the strains *Aspergillus* sp. St1, *P. javanicum* St2, St8 and *F. oxysporum* St6, St7 exhibited visible growth. Moreover, the substantial utilization of tetradecane suggested the strains’ superior bioconversion ability to degrade tetradecane.

Despite the early description of *Penicillium javanicum*, which has the homotypic synonym *Eupenicillium javanicum*, in 1929 [29], followed by its isolation from various habitats [30,31,32,33,34,35], little is known about the utilization of petroleum hydrocarbons by this species. In one study by Oudot et al. [36], various strains of tropical fungi, including *E. javanicum* strains, were isolated from petroleum-contaminated soil and river sediments and analyzed for the degradation of oil and oil fractions. *E. javanicum* strains were among the active degraders of oil and fractions of saturated and aromatic hydrocarbons. However, detailed investigations into the degradation pathways or studies concerning their ability to use specific *n*-alkanes were not further described. Very recently, two *P. javanicum* isolates from oil-contaminated soils showed strong growth and almost complete degradation of tetradecane [12], which is comparable to the behavior of *P. javanicum* St2 and St8 in this study.

*F. oxysporum* was first introduced by Diederich F.L. von Schlechtendal in 1824. It harbors both pathogenic and non-pathogenic strains, suffers from various classification problems, and was established as an epitype of *F. oxysporum* by Lombard et al. in 2019 [37]. These are probably also the reasons why there are far-reaching fields of research on this species, but very little on the biodegradation of alkanes. *F. oxysporum* strains were only described as tetradecane [14] or pristane (a branched chain alkane) [12] degraders. More frequently, *F. oxysporum* strains have been described as utilizers of aromatics or polycyclic aromatic hydrocarbons [38,39,40,41,42] or as strains isolated on these hydrocarbons or on crude oil [43,44].

The genus *Aspergillus*, already described in 1729 [45], comprises more than 400 species. This number is constantly increasing, and the genus has been repeatedly taxonomically revised over the years [46,47,48,49,50,51,52,53,54], which also explains the variety of studies of this genus on the biodegradation of alkanes. Due to these facts, it is only possible to consider a selected part of this work in connection with our results on the strain *Aspergillus* sp. St1. Very comparable results to our strain, *Aspergillus* sp. St1, could be demonstrated with *Aspergillus* sp. and *Aspergillus niger* strains regarding the turnover of hexadecane [55,56,57]. High degradation of *n*-alkanes, a strong increase in biomass and a better turnover in static cultures compared to rotating cultures were described. The *Aspergillus niger* and *A. terreus* strains were able to form high amounts of mycelial biomass growing on *n*-alkanes C_13_ to C_18_ and showed high utilization of all of these substrates [58]. Furthermore, various *Aspergillus* strains were able to utilize the *n*-alkane fraction of crude oil very well, with chain length ranging from C_8_ in octane to C_30_ in triacontane [59], and also crude oil without differentiation of fractions [58,60].

Among the potential tetradecane degraders investigated in this study, *T. harzianum* St3 showed the poorest increase in the biomass and the highest concentration of remaining substrate. A previous study had confirmed the differential response of *T. harzianum* compared to *Aspergillus* sp. and *F. oxysporum* [61]. The genera were tested for their ability to survive after increasing the crude oil concentration from 1% to 5%. With 1% crude oil, all genera were still viable. However, when 5% crude oil was added, only *Trichoderma* could no longer be detected [61]. This aligns with the poor growth and hence the scarce substrate utilization of *T. harzianum* St3 observed in the current study. On the other hand, various scientific literatures have extensively characterized *T. harzianum* strains as efficacious bioremediators of oil contaminants [62,63,64,65]. The current scientific consensus reflects a need for further research to robustly establish the effectiveness of *T. harzianum* as a bioremediator for oil-derived compounds.

**Figure 6:**
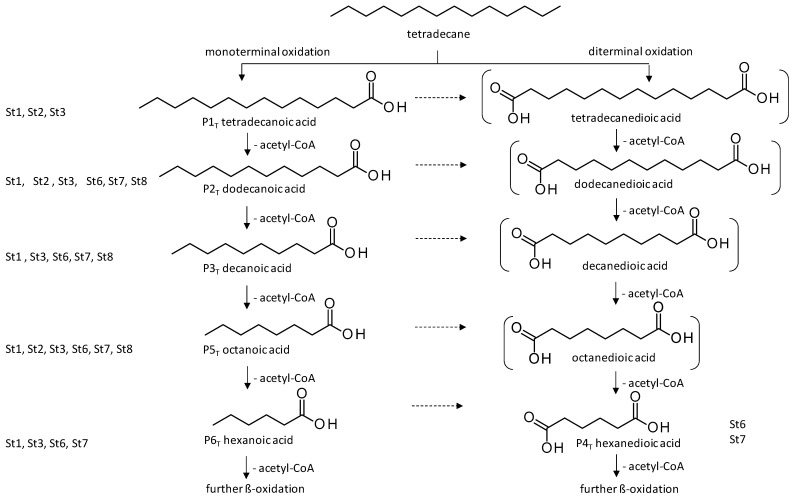
Monoterminal and diterminal degradation pathways of tetradecane by *Aspergillus* sp. St1, *P. javanicum* St2, St8, *T. harzianum* St3, and *F. oxysporum* St6, St7. Structures that were not detected in the current study are marked by brackets. Pn_T_ refers to the products’ number as per its appearance in Table 2 and Table 3.

The degradation of tetradecane can occur via monoterminal and diterminal degradation mechanisms. Metabolites detected in the acidic extracts in this study indicated monoterminal degradation pathways for all strains, except for those detected in the cultures of *F. oxysporum* strains St6 and St7 that supported in addition a diterminal degradation mechanism (Figure 6). The monoterminal oxidation started at one terminal methyl group of tetradecane, which was first hydroxylated to the primary alcohol, then dehydrogenated to aldehyde and subsequently oxidized to the monocarboxylic fatty acid [12,18,19,26,66,67]. It is known that a cytochrome P450 monooxygenase bound in the fungal cell membrane acts as the key enzyme for the first oxidative hydroxylation attack by microorganisms [18,19,66,67,68]. The resulting tetradecanoic acid then served as a substrate for acyl-CoA synthetase, which produced acyl-CoA. This was then cleaved through β-oxidation, and new saturated fatty acids with shorter chains were formed (Figure 6; [18,19,66]). *F. oxysporum* went a step further in complexity by additionally executing diterminal degradation, where both terminal methyl groups were hydroxylated. Similar to monoterminal oxidation, the initial step of hydroxylation requires the activity of cytochrome P450 monooxygenase [18,66,67,68]. The resulting dialdehyde then degraded to dicarboxylic acids (i.e., hexanedioic acid), and cleavage of acyl-CoA through β-oxidation occurred. Dodecanoic acid could be detected in all strains, with further stepwise cleavage of acyl-CoA to decanoic (except for *P. javanicum* St2), followed by the formation of octanoic acid (in all strains) and to hexanoic acid (except for *P. javanicum* St2 and St8). The obtained degradation profiles mirror the typical fungal metabolic traits when exposed to paraffinic substrates [69].

### 4.2. Consumption and Degradation of Cyclohexanone and Cyclohexane

**Figure 7:**
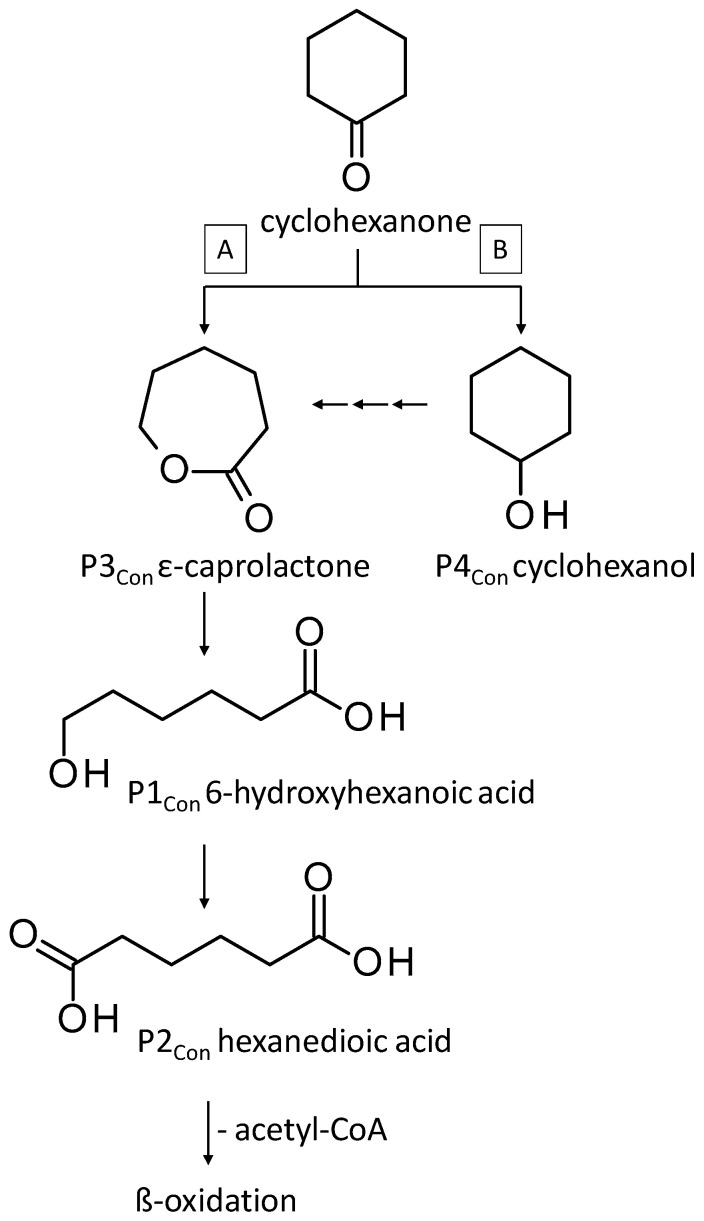
Degradation pathways of cyclohexanone by *F. oxysporum* St4 and St5 compiled from pathways of Morgan and Watkinson [75] and Mandal et al. [76]. Pn_con_ refers to the products’ number as per its appearance in Table 4.

Cyclohexanone and cyclohexane are alicyclic crude oil components that are described to be resistant to degradation by microorganisms due to their membrane toxicity and low water solubility [14,27,68,69,70,71,72,73]. As recently as 2019, Prenafeta-Boldú et al. wrote that the literature on the fungal metabolism of alicyclic compounds is almost non-existent [18]. In addition to some previously described studies [14,19,27,74] and in order to complement research in this field, cyclohexanone was used as an isolation substrate in this study. The biodegradation capacity of the two *F. oxysporum* strains St4 and St5 was evaluated in the presence of cyclohexanone, which was degraded via ε-caprolactone, 6-hydroxyhexanoic and hexanedioic acid (Figure 7, pathway A) following the classical descriptions of bacterial cyclohexane degradation via cyclohexanone [75]. The degradation of cyclohexanone by our two isolated filamentous fungi involved enzyme-catalyzed reactions, particularly the Baeyer–Villiger reaction, which used cyclohexanone monooxygenase to convert cyclohexanone into ε-caprolactone, a cyclic ester. This ester would be converted into 6-hydroxyhexanoic acid and further oxidized to hexanedioic acid, which can be further metabolized by ß-oxidation. Comparable degradations have also been described for some other filamentous fungi of the genus *Fusarium* and also for yeasts; e.g., for the genus *Rhodotorula* [14,76]. Quite surprisingly, one of the metabolites of cyclohexanone degradation is cyclohexanol (Table 4, Figure 7 pathway B), also described for other *Fusarium* and *Rhodotorula* strains [14,76], whereby it was even demonstrated for one *Fusarium* sp. strain that cyclohexanone is first degraded to cyclohexanol and only then ε-caprolactone can be detected [76] (Figure 7, pathway B). This temporal sequence of product formation could not be demonstrated for our two strains of *F. oxysporum* St4 and St5, which lead to the assumption that both degradation pathways A and B are conceivable.

**Figure 8:**
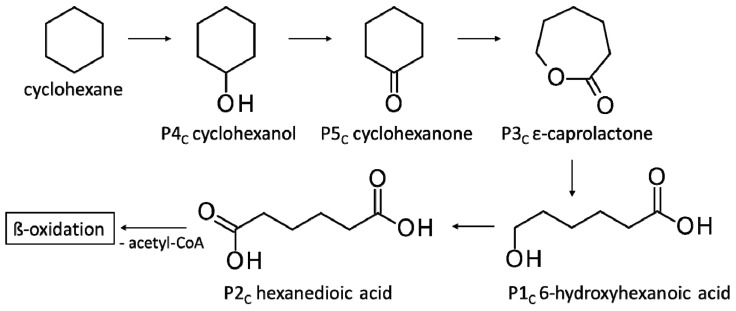
Degradation pathways of cyclohexane by *F. oxysporum* St4. Pn_C_ refers to the products’ number as per its appearance in Table 5.

Since incubations of *F. oxysporum* St4 and St5 behaved similarly, indicating visible substrate degradation potential of cyclohexanone, St4 was further tested as an example for the degradation potential of cyclohexane, the unoxidized parent compound of cyclohexanone. The aim was to test whether this strain can completely metabolize a non-oxidized alicyclic parent compound if introduced as a starter substrate. *F. oxysporum* St4 was able to metabolize also cyclohexane, which, to our knowledge, is the first time describing this property for this species. The identified metabolites were cyclohexanol, cyclohexanone, ε-caprolactone, 6-hydroxyhexanoic and hexanedioic acid (Figure 8), which corresponded to those for the metabolism of cyclohexanone (Figure 7). The microbial degradation of cyclohexane has been reported for years as co-metabolism, in microbial communities or as co-metabolic process with mixed cultures [73,75].

In this context, the study by Dallinger et al. [27] demonstrated the ability of yeasts to use cyclohexane by initial hydroxylation and dehydrogenation, but no further conversion was possible. Cyclohexanol and cyclohexanone were detected as the only products with no further degradation of cyclohexanone. Methylcyclohexane and longer *n*-alkylcyclohexanes, on the other hand, can be completely degraded via side chain degradation, aromatization and aromatic ring cleavage reactions [19,74]. However, both *Fusarium* strains St4 and St5 presented in this work were able to metabolize cyclohexanone to ε-caprolactone demonstrating that cyclohexanone is not a dead-end product. The same was further confirmed after testing the ability of *F. oxysporum* St4 to degrade cyclohexane (Figure 8), which illustrates that a complete degradation of alicyclic compounds like cyclohexane by fungal strains, in this case a filamentous fungus, is possible.

Together with the ability of *F. oxysporum* St6 and St7 to degrade tetradecane (Section 4.1), and the biodegradation ability of St4 and St5 to degrade alicyclic substrates highlighted *F. oxysporum* as a specialist, capable of degrading a wider variety of hydrocarbons. These findings agree with the literature that cast the *Fusarium* genus as a formidable degrader of various hydrocarbons [12,14,77].

## 5. Conclusions

On the basis of all degradation results presented, we conclude that tetradecane, cyclohexanone and cyclohexane, all model components of the Kazakh crude oil, can be degraded by the eight filamentous fungi. Thus, this article offers a solid basis for the mycoremediation abilities of the selected isolates, which can be further supported by a comparative in situ analysis of their native habitat.

For mycoremediation, an important contribution to the degradation of pollutants is attributed to these microorganisms, suggesting that these newly isolated species might be used for remediation projects in Kazakhstan and elsewhere.

## Figures and Tables

**Table 1:** List of the eight fungal isolates tested in the current study.

Isolate *	Scientific Name **	Abbreviation	Soil Source	Isolation Substrate
SBUG-M1743	*Aspergillus* sp.	St1	Park near Almaty train station	Tetradecane
SBUG-M1744	*Penicillium javanicum*	St2	Oil deposit “Aktöbe”	
SBUG-M1750	*Trichoderma harzianum*	St3	Park near Almaty train station	
SBUG-M1746	*Fusarium oxysporum*	St4	Oil depot “Ozen”	Cyclohexanone
SBUG-M1748	*Fusarium oxysporum*	St5	Park near Almaty train station	
SBUG-M1768	*Fusarium oxysporum*	St6	Oil deposit “Aktöbe”	Crude oil
SBUG-M1769	*Fusarium oxysporum*	St7		
SBUG-M1770	*Penicillium javanicum*	St8		

* SBUG stands for the strain collection of the Department of Biology at the University of Greifswald at the Institute for Microbiology. ** Strains identified based in their ITS sequences as described in Table S1.

**Table 2:** Detected acids formed during the biodegradation of tetradecane by *Aspergillus* sp. St1, *P. javanicum* St2 and *T. harzianum* St3.

Products		Retention Time (min)	St1		St2		St3	
			Type of Culture					
			Shake	Static	Shake	Static	Shake	Static
P1_T_	Tetradecanoic acid 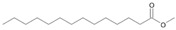	32.50	+	+	+	+	+	+
P2_T_	Dodecanoic acid 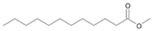	26.87	+	+	+	+	+	+
P3_T_	Decanoic acid 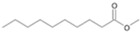	20.66	+	+	-	-	-	+
P4_T_	Hexanedioic acid 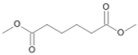	18.20	-	-	-	-	-	-
P5_T_	Octanoic acid 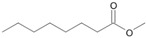	13.94	+	+	+	+	-	+
P6_T_	Hexanoic acid 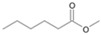	7.22	+	+	-	-	+	-

+ detectable; - non-detectable. Pn_T_ refers to the biodegradation products (P) of tetradecane (T). Products were detected in the acidic methylated extracts.

**Table 3:** Detected acids formed during the biodegradation of tetradecane by *F. oxysporum* St6, St7 and *P. javanicum* St8.

Products		Retention Time (min)	St6	St7	ST8
P1_T_	Tetradecanoic acid 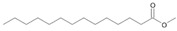	32.50	-	-	-
P2_T_	Dodecanoic acid 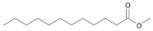	26.87	+	+	+
P3_T_	Decanoic acid 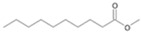	20.66	+	+	+
P4_T_	Hexanedioic acid 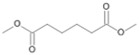	18.20	+	+	-
P5_T_	Octanoic acid 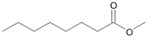	13.94	+	+	+
P6_T_	Hexanoic acid 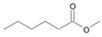	7.22	+	+	-

+ detectable; - non-detectable. Pn_T_ refers to the biodegradation products (P) of tetradecane (T). Incubations were conducted under shaking conditions and products were detected in the acidic methylated extracts.

**Table 4:** Detected products formed during the biodegradation of cyclohexanone by *F. oxysporum* St4 and St5.

Products		Retention Time (min)	St4		St5	
			Type of Culture			
			Shake	Static	Shake	Static
P1_Con_	6-Hydroxyhexanoic acid * 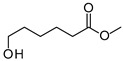	22.4 *	+	-	+	-
P2_Con_	Hexanedioic acid * 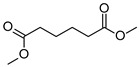	18.2 *	+	+	+	+
P3_Con_	ε-Caprolactone ** 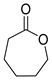	8.7 **	+	+	+	+
P4_Con_	Cyclohexanol ** 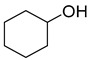	4.4 **	+	+	+	+

+ detectable; - non-detectable. * detected in acidic methylated extracts; ** detected in alkaline extracts. Pn_Con_ refers to the biodegradation products (P) of cyclohexanone (Con). P2_Con_ is the equivalent product to P4_T_ and P2_C_ that are listed in Table 2, Table 3 and Table 5, respectively.

**Table 5:** Detected products formed during the biodegradation of cyclohexane by *F. oxysporum* St4.

Products		Retention Time (min)	Shake	Static
P1_C_	6-Hydroxyhexanoic acid * 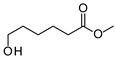	22.4 *	+	+
P2_C_	Hexanedioic acid * 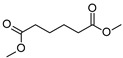	18.2 *	+	+
P3_C_	ε-Caprolactone ** 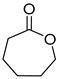	8.7 **	+	+
P4_C_	Cyclohexanol ** 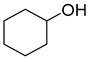	4.4 **	+	+
P5_C_	Cyclohexanone ** 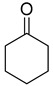	4.3 **	+	+

+ detectable; * detected in acidic methylated extracts; ** detected in alkaline extracts. Pn_C_ refers to the biodegradation products (P) of cyclohexane (C). P2_C_ is the equivalent product to P4_T_ and P2_Con_ that are listed in Table 2, Table 3 and Table 4, respectively.

## Data Availability

Data are contained within the article and Supplementary Materials.

## Supplementary Materials

The following supporting information can be downloaded at https://www.mdpi.com/article/10.3390/jof10060436/s1, Figure S1: Macroscopical views of filamentous fungi grown on MAg plates for 7 days. (a) SBUG-M1743, (b) SBUG-M1744, (c) SBUG-M1750, (d) SBUG-M1746, (e) SBUG-M1748, (f) SBUG-M1768, (g) SBUG-M1769 and (h) SBUG-M1770; Figure S2: Microscopic images of the isolated strains after 3 and 7 days of growth. (a) SBUG-M1743, (b) SBUG-M1744, (c) SBUG-M1750, (d) SBUG-M1746, (e) SBUG-M 748. Magnification power is 400× except for ‘a’ is 200×; Figure S3: Microscopic images of the isolated strains after 7 days of growth. (a,b) SBUG-M 1768, (c,d) SBUG-M1769, and (e,f) SBUG-M1770. Magnification power is 400×. Figure S4: Remained substrate after incubating six fungal strains with 0.25% tetradecane for 7 days in shake cultures. *Aspergillus* sp. SBUG-M1743, *P. javanicum* SBUG-M1744, and *T. harzianum* SBUG-M1750 were further tested for their growth and degradation abilities with 0.5% tetradecane (Figure 2); Figure S5: Macroscopical features of cultures incubated with tetradecane and their controls after 7 days at 30 °C. (a,b) *Aspergillus* sp. SBUG-M1743, (c,d) *P javanicum* SBUG-M1744, and (e) *T. harzianum* SBUG-M1750; Figure S6: Macroscopical features of *F. oxysporum* cultures incubated with cyclohexanone and their controls after 7 days at 30 °C. (a,b) SBUG-M1746, and (c,d) SBUG-M 1748. Table S1: Results of identification of the isolated filamentous fungi by ITS gene sequence analysis in NCBI- ITS database; Table S2: Macroscopic characterization of the filamentous fungi after 7 days of growth on MAg medium; Table S3: Growth of SBUG-M1743, SBUG-M1744, SBUG-M1750, SBUG-M1768, SBUG-M1769 and SBUG-M1770 on their isolation substrate tetradecane; Table S4: Growth of SBUG-M1746, and SBUG-M1748 on their isolation substrate cyclohexanone.
