# Supplementary material for: Comparative Bioremediation of Tetradecane, Cyclohexanone and Cyclohexane by Filamentous Fungi from Polluted Habitats in Kazakhstan
Source: J Fungi (Basel). 2024 Jun 19;10(6):436. doi: 10.3390/jof10060436 (PMC11204954; doi:10.3390/jof10060436)
Supplement: Supplementary file 1 [file jof-10-00436-s001.zip › jof-3023767-supplementary.pdf]

Journal of *Fungi*

Supplementary materials

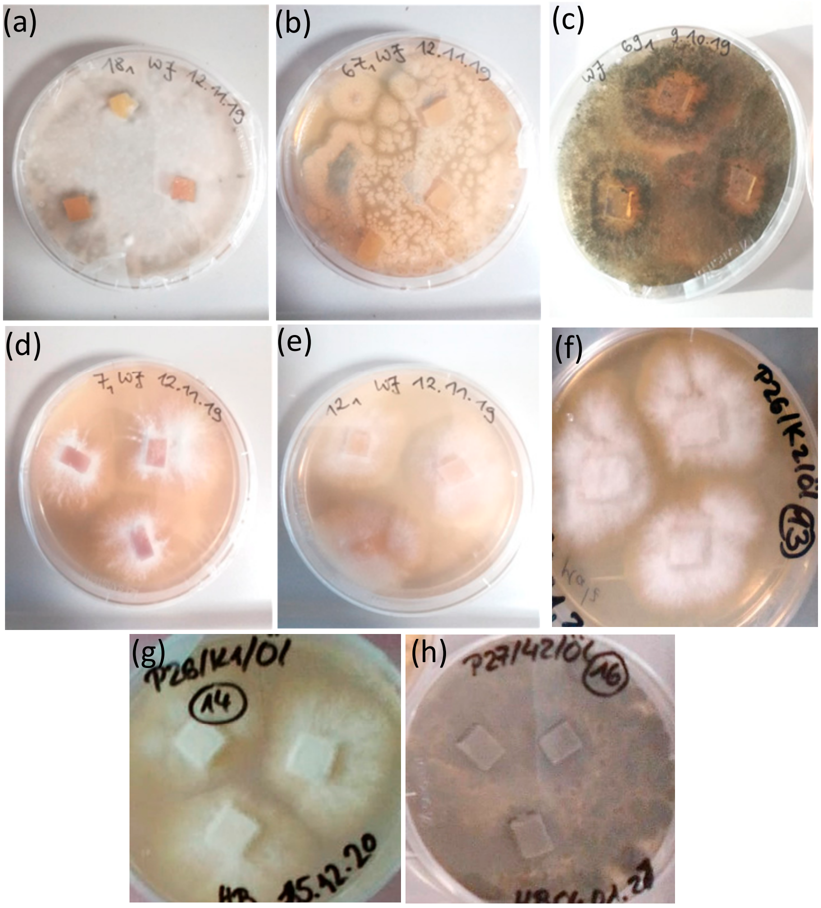

Figure S1. Macroscopical views of filamentous fungi grown on MAg plates for 7 days. (a) SBUG-M1743, (b) SBUG-M1744, (c) SBUG-M1750, (d) SBUG-M1746, (e) SBUG-M1748, (f) SBUG-M1768, (g) SBUG-M1769 and (h) SBUG-M1770. Inoculations were done via three-point method.

Journal of Fungi

MDPI

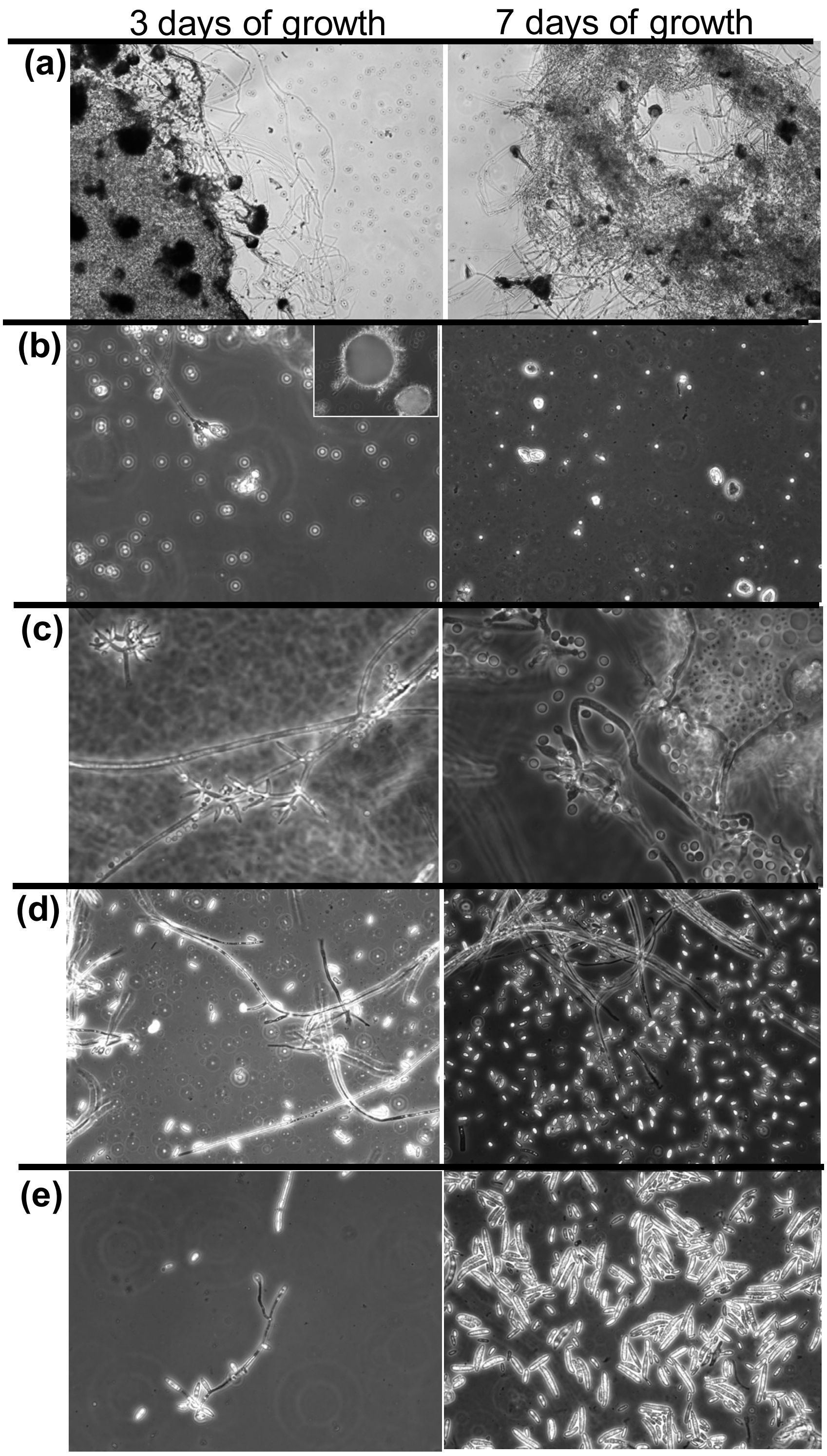

Figure S2. Microscopic images of the isolated strains after 3 and 7 days of growth. (a) SBUG-M1743, (b) SBUG-M1744, (c) SBUG-M1750, (d) SBUG-M1746, and (e) SBUG-M 748. Magnification power is 400x except for ‚a' is 200x.

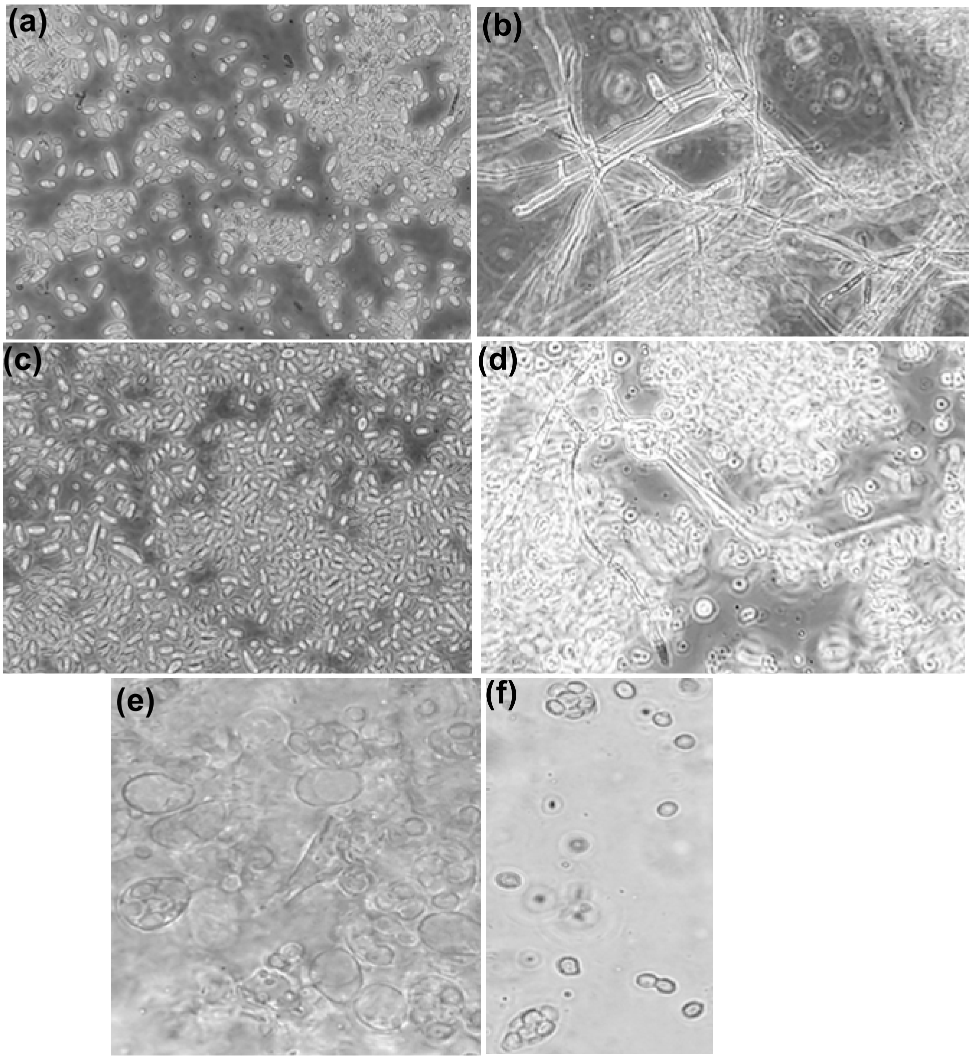

Figure S3. Microscopic images of the isolated strains after 7 days of growth. (a, b) SBUG-M 1768, (c, d) SBUG-M1769, and (e, f) SBUG-M1770. Magnification power is 400x.

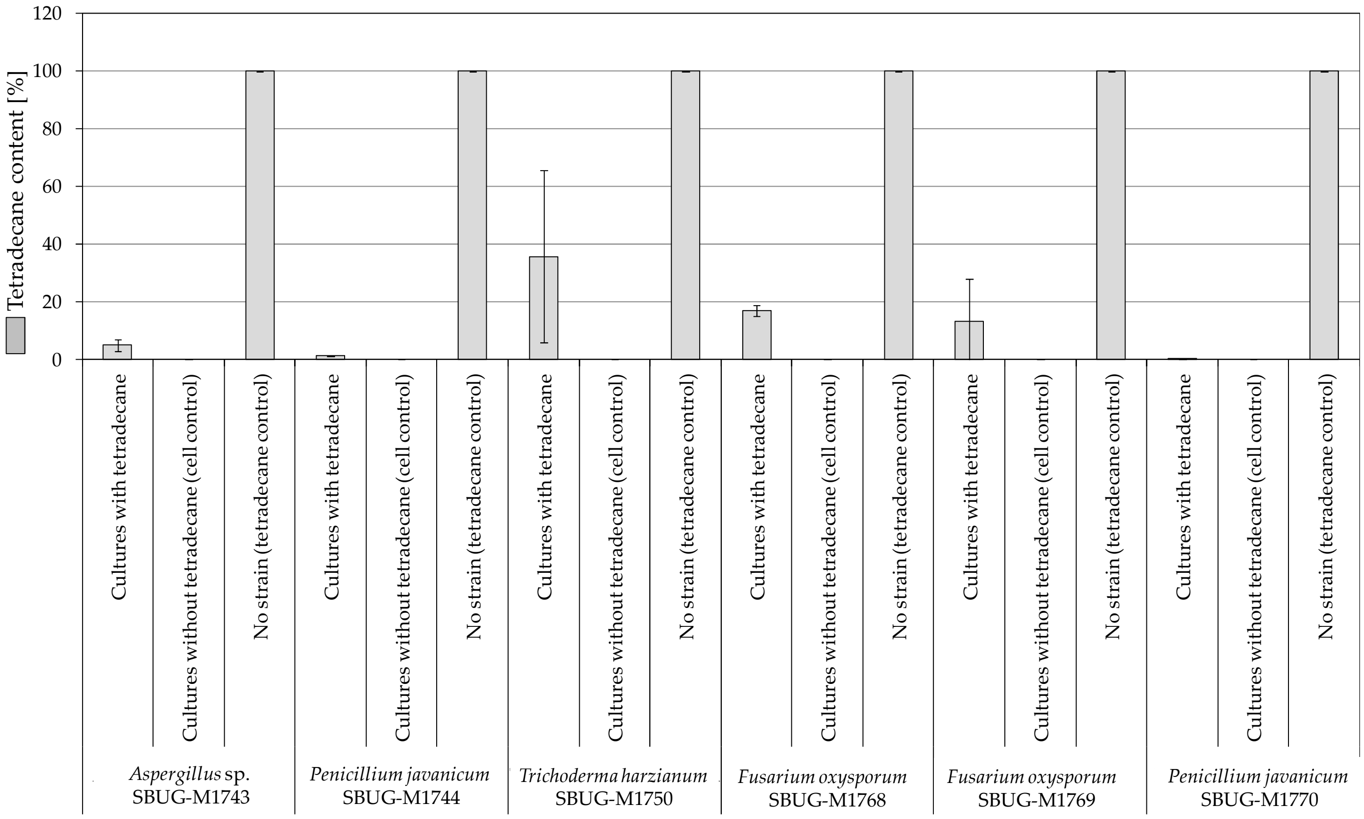

Figure S4. Remained substrate after incubating six fungal strains with 0.25% tetradecane for 7 days in shake cultures. *Aspergillus* sp. SBUG-M1743, *P. javanicum* SBUG-M1744, and *T. harzianum* SBUG-M1750 were further tested for their growth and degradation abilities with 0.5% tetradecane (Figure 2).

Journal of Fungi

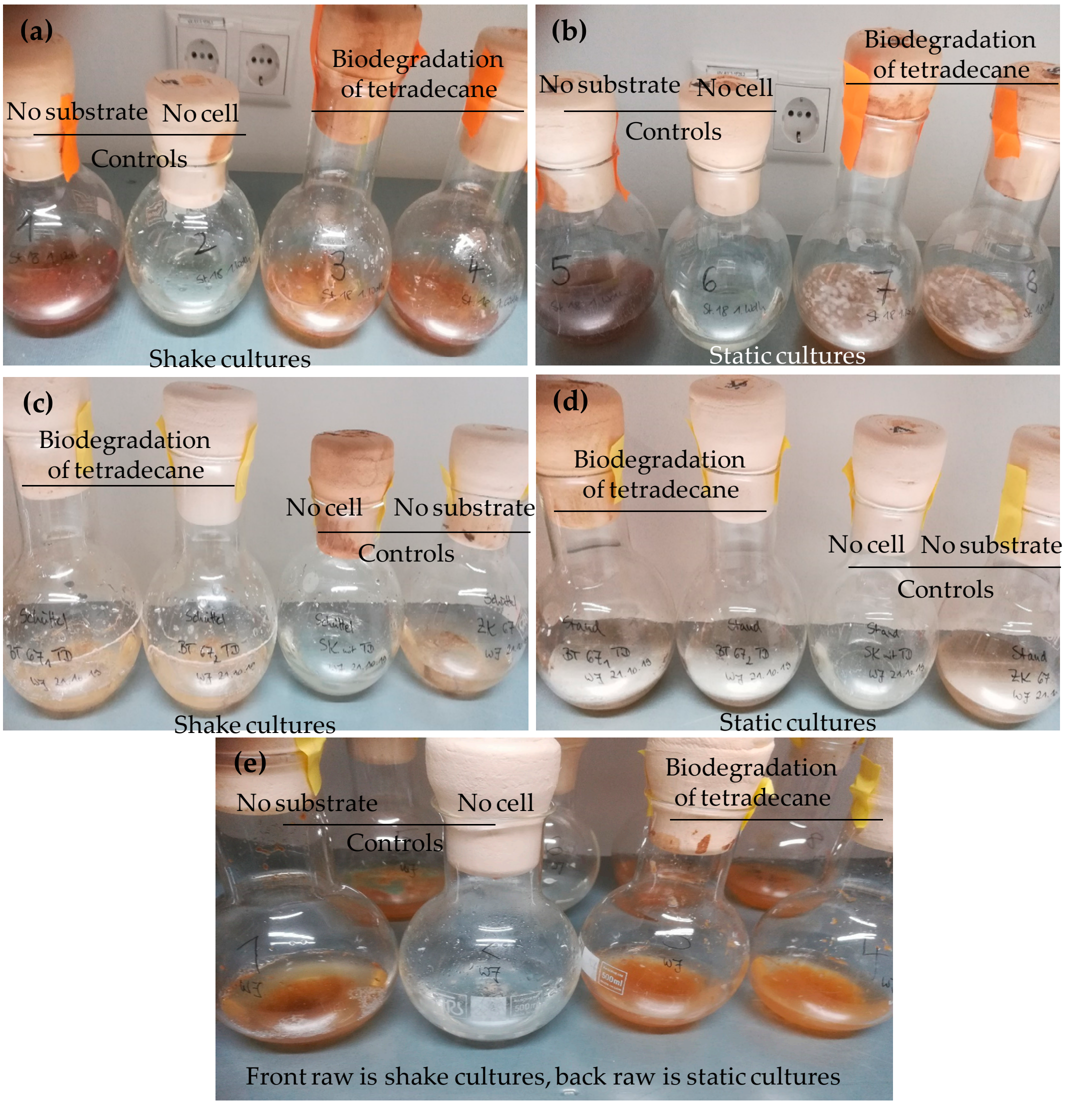

Figure S5: Macroscopical features of cultures incubated with tetradecane and their controls after 7 days at 30°C. (a, b) *Aspergillus* sp. SBUG-M1743, (c, d) *P. javanicum* SBUG-M1744, and (e) *T. harzianum* SBUG-M1750.

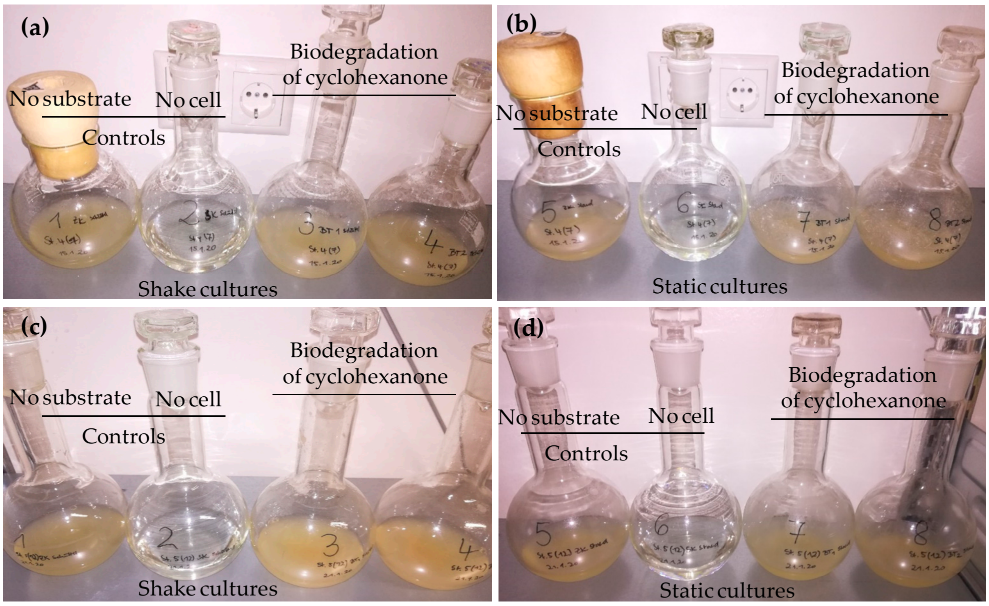

Figure S6: Macroscopical features of *F. oxysporum* cultures incubated with cyclohexanone and their controls after 7 days at 30°C. (a, b) SBUG-M1746, and (c, d) SBUG-M1748.

Table S1. Results of identification of the isolated filamentous fungi by ITS gene sequence analysis in NCBI- ITS database.

Isolate	Result of identification [b)]	ITS gene sequence analysis [a)]					
		**Description**	**Max score**	**Total score**	**Query cover**	**Identity**	**Accession N°**
**SBUG-M1743**	***Aspergillus* sp.**	*Aspergillus fumigatus* strain P12 internal transcribed spacer 1, partial sequence; 5.8S ribosomal RNA gene and internal transcribed spacer 2, complete sequence; and large subunit ribosomal RNA gene, partial sequence	1164	1653	99%	96.74%	OP681422.1
		Aspergillus fumigatus isolate MEBP0074 small subunit ribosomal RNA gene, partial sequence; internal transcribed spacer 1, 5.8S ribosomal RNA gene, and internal transcribed spacer 2, complete sequence; and large subunit ribosomal RNA gene, partial sequence	1147	1597	99%	96.56%	MT597427.1
		Aspergillus fumigatus isolate DBB5 small subunit ribosomal RNA gene, partial sequence; internal transcribed spacer 1, 5.8S ribosomal RNA gene, and internal transcribed spacer 2, complete sequence; and large subunit ribosomal RNA gene, partial sequence	1122	1122	92%	96%	OM218663.1
		Aspergillus fumigatus isolate MEBP0063 small subunit ribosomal RNA gene, partial sequence; internal transcribed spacer 1, 5.8S ribosomal RNA gene, and internal transcribed spacer 2, complete sequence; and large subunit ribosomal RNA gene, partial sequence	1120	1360	99%	95.60%	MT591987.1
		Aspergillus aureolus isolate KNU14-7 18S ribosomal RNA gene, partial sequence	1120	1606	98%	99.04%	KP966615.1
		Aspergillus fumigatus isolate DGGE gel band small subunit ribosomal RNA gene, partial sequence; internal transcribed spacer 1, 5.8S ribosomal RNA gene, and internal transcribed spacer 2, complete sequence; and large subunit ribosomal RNA gene, partial sequence	1109	1554	99%	96.60%	MN520104.1
		Aspergillus udagawae strain det 18.042M small subunit ribosomal RNA gene, partial sequence; internal transcribed spacer 1, 5.8S ribosomal RNA gene, and internal transcribed spacer 2, complete sequence; and large subunit ribosomal RNA gene, partial sequence	1086	1308	100%	99.34%	MN882827.1
		Aspergillus udagawae strain CBS DTO_283-D3 small subunit ribosomal RNA gene, partial sequence; internal transcribed spacer 1, 5.8S ribosomal RNA gene, and internal transcribed spacer 2, complete sequence; and large subunit ribosomal RNA gene, partial sequence	1086	1308	100%	99.34%	KY808744.1
		Aspergillus aureolus strain CBS DTO_278-B7 small subunit ribosomal RNA gene, partial sequence; internal transcribed spacer 1, 5.8S ribosomal RNA gene, and internal transcribed spacer 2, complete sequence; and large subunit ribosomal RNA gene, partial sequence	1086	1308	100%	99.34%	KY808743.1

Table S1 (continue)

		Aspergillus udagawae strain CBS DTO_166-D6 small subunit ribosomal RNA gene, partial sequence; internal transcribed spacer 1, 5.8S ribosomal RNA gene, and internal transcribed spacer 2, complete sequence; and large subunit ribosomal RNA gene, partial sequence	1086	1308	100%	99.34 %	KY808740.1
**SBUG-M1744**	***Penicillium javanicum***	*Penicillium caperatum* isolate CMV001H1 18S ribosomal RNA gene, partial sequence; internal transcribed spacer 1, 5.8S ribosomal RNA gene, and internal transcribed spacer 2, complete sequence; and 28S ribosomal RNA gene, partial sequence	1029	1029	100%	99.82%	MK450677.1
		Penicillium caperatum isolate CMV013G2 18S ribosomal RNA gene, partial sequence; internal transcribed spacer 1, 5.8S ribosomal RNA gene, and internal transcribed spacer 2, complete sequence; and 28S ribosomal RNA gene, partial sequence	1029	1029	100%	99.82%	MK951937.1
		Penicillium javanicum culture CBS:129395 strain CBS 129395 small subunit ribosomal RNA gene, partial sequence; internal transcribed spacer 1, 5.8S ribosomal RNA gene, and internal transcribed spacer 2, complete sequence; and large subunit ribosomal RNA gene, partial sequence	1029	1029	100%	99.82%	MH865296.1
		Penicillium javanicum culture CBS:291.53 strain CBS 291.53 small subunit ribosomal RNA gene, partial sequence; internal transcribed spacer 1, 5.8S ribosomal RNA gene, and internal transcribed spacer 2, complete sequence; and large subunit ribosomal RNA gene, partial sequence	1029	1029	100%	99.82%	MH857207.1
		Penicillium javanicum strain WTS27 18S ribosomal RNA gene, partial sequence; internal transcribed spacer 1, 5.8S ribosomal RNA gene, and internal transcribed spacer 2, complete sequence; and 28S ribosomal RNA gene, partial sequence	1029	1029	100%	99.82%	GU966506.1
		Penicillium meloforme clone EF_622 internal transcribed spacer 1, partial sequence; 5.8S ribosomal RNA gene and internal transcribed spacer 2, complete sequence; and large subunit ribosomal RNA gene, partial sequence	1027	1027	99%	99.82%	MT529271.1
		Penicillium meloforme clone SF_297 internal transcribed spacer 1, partial sequence; 5.8S ribosomal RNA gene and internal transcribed spacer 2, complete sequence; and large subunit ribosomal RNA gene, partial sequence	1026	1026	99%	99.82%	MT529573.1
		Penicillium meloforme clone EF_621 internal transcribed spacer 1, partial sequence; 5.8S ribosomal RNA gene and internal transcribed spacer 2, complete sequence; and large subunit ribosomal RNA gene, partial sequence	1024	1024	99%	99.82%	MT529270.1

Table S1 (continue)

		Penicillium caperatum isolate CMV012C4 18S ribosomal RNA gene, partial sequence; internal transcribed spacer 1, 5.8S ribosomal RNA gene, and internal transcribed spacer 2, complete sequence; and 28S ribosomal RNA gene, partial sequence	1024	1024	100%	99.64%	MK450678.1
		Penicillium javanicum culture CBS:129771 strain CBS 129771 small subunit ribosomal RNA gene, partial sequence; internal transcribed spacer 1, 5.8S ribosomal RNA gene, and internal transcribed spacer 2, complete sequence; and large subunit ribosomal RNA gene, partial sequence	1024	1024	100%	99.64%	MH865649.1
**SBUG-M1750**	***Trichoderma harzianum***	*Trichoderma* sp. isolate S-6-1-1 internal transcribed spacer 1, partial sequence; 5.8S ribosomal RNA gene and internal transcribed spacer 2, complete sequence; and large subunit ribosomal RNA gene, partial sequence	1048	1048	100%	100%	MT133832.1
		Trichoderma harzianum isolate HCH small subunit ribosomal RNA gene, partial sequence; internal transcribed spacer 1, 5.8S ribosomal RNA gene, and internal transcribed spacer 2, complete sequence; and large subunit ribosomal RNA gene, partial sequence	1048	1048	100%	100%	MK793273.1
		Trichoderma harzianum isolate EF07 internal transcribed spacer 1, partial sequence; 5.8S ribosomal RNA gene and internal transcribed spacer 2, complete sequence; and large subunit ribosomal RNA gene, partial sequence	1048	1048	100%	100%	MN658590.1
		Trichoderma harzianum strain T2B1 internal transcribed spacer 1, partial sequence; 5.8S ribosomal RNA gene and internal transcribed spacer 2, complete sequence; and large subunit ribosomal RNA gene, partial sequence	1048	1048	100%	100%	MN518418.1
		Trichoderma harzianum isolate DA11 internal transcribed spacer 1, partial sequence; 5.8S ribosomal RNA gene and internal transcribed spacer 2, complete sequence; and large subunit ribosomal RNA gene, partial sequence	1048	1048	100%	100%	MN396739.1
		Fungal sp. isolate K_MISO2_3_16 internal transcribed spacer 1, partial sequence; 5.8S ribosomal RNA gene and internal transcribed spacer 2, complete sequence; and large subunit ribosomal RNA gene, partial sequence	1048	1048	100%	100%	MK594783.1

Table S1 (continue)

		Trichoderma sp. isolate SDAS203177 small subunit ribosomal RNA gene, partial sequence; internal transcribed spacer 1, 5.8S ribosomal RNA gene, and internal transcribed spacer 2, complete sequence; and large subunit ribosomal RNA gene, partial sequence	1048	1048	100%	100.%	MK870388.1
		Trichoderma sp. isolate SDAS203451 internal transcribed spacer 1, partial sequence; 5.8S ribosomal RNA gene and internal transcribed spacer 2, complete sequence; and large subunit ribosomal RNA gene, partial sequence	1048	1048	100%	100%	MK870236.1
		Trichoderma sp. isolate SDAS203158 small subunit ribosomal RNA gene, partial sequence; internal transcribed spacer 1, 5.8S ribosomal RNA gene, and internal transcribed spacer 2, complete sequence; and large subunit ribosomal RNA gene, partial sequence	1048	1048	100%	100%	MK870185.1
		Trichoderma sp. isolate SDAS203157 small subunit ribosomal RNA gene, partial sequence; internal transcribed spacer 1, 5.8S ribosomal RNA gene, and internal transcribed spacer 2, complete sequence; and large subunit ribosomal RNA gene, partial sequence	1048	1048	100%	100%	MK870184.1
**SBUG-M1746**	***Fusarium oxysporum***	*Fusarium oxysporum* f. sp. ciceris isolate FOCAMU5 internal transcribed spacer 1, partial sequence; 5.8S ribosomal RNA gene and internal transcribed spacer 2, complete sequence; and large subunit ribosomal RNA gene, partial sequence	850	850	100%	100%	PP217446.1
		Fusarium oxysporum isolate AK2 small subunit ribosomal RNA gene, partial sequence; internal transcribed spacer 1, 5.8S ribosomal RNA gene, and internal transcribed spacer 2, complete sequence; and large subunit ribosomal RNA gene, partial sequence	850	850	100%	100%	OR915474.1
		Fusarium foetens isolate P101D_F internal transcribed spacer 1, partial sequence; 5.8S ribosomal RNA gene and internal transcribed spacer 2, complete sequence; and large subunit ribosomal RNA gene, partial sequence	850	850	100%	100%	OR905647.1

Table S1 (continue)

Fusarium foetens isolate M81B_F internal transcribed spacer 1, partial sequence; 5.8S ribosomal RNA gene and internal transcribed spacer 2, complete sequence; and large subunit ribosomal RNA gene, partial sequence	850	850	100%	100%	OR905620.1
Fusarium oxysporum isolate F240 small subunit ribosomal RNA gene, partial sequence; internal transcribed spacer 1, 5.8S ribosomal RNA gene, and internal transcribed spacer 2, complete sequence; and large subunit ribosomal RNA gene, partial sequence	850	850	100%	100.00%	OR123360.1
Fusarium oxysporum isolate F169 small subunit ribosomal RNA gene, partial sequence; internal transcribed spacer 1, 5.8S ribosomal RNA gene, and internal transcribed spacer 2, complete sequence; and large subunit ribosomal RNA gene, partial sequence	850	850	100%	100%	OR123329.1
Fusarium oxysporum isolate F127 internal transcribed spacer 1, partial sequence; 5.8S ribosomal RNA gene and internal transcribed spacer 2, complete sequence; and large subunit ribosomal RNA gene, partial sequence	850	850	100%	100%	OR123312.1
Fusarium oxysporum isolate F91 small subunit ribosomal RNA gene, partial sequence; internal transcribed spacer 1, 5.8S ribosomal RNA gene, and internal transcribed spacer 2, complete sequence; and large subunit ribosomal RNA gene, partial sequence	850	850	100%	100%	OR123300.1
Fusarium oxysporum isolate F17 small subunit ribosomal RNA gene, partial sequence; internal transcribed spacer 1, 5.8S ribosomal RNA gene, and internal transcribed spacer 2, complete sequence; and large subunit ribosomal RNA gene, partial sequence	850	850	100%	100%	OR123279.1
Fusarium nirenbergiae isolate FS89 Broca INT small subunit ribosomal RNA gene, partial sequence; internal transcribed spacer 1, 5.8S ribosomal RNA gene, and internal transcribed spacer 2, complete sequence; and large subunit ribosomal RNA gene, partial sequence	850	850	100%	100%	OR538698.1
Fusarium oxysporum isolate YBC01 small subunit ribosomal RNA gene, partial sequence; internal transcribed spacer 1, 5.8S ribosomal RNA gene, and internal transcribed spacer 2, complete sequence; and large subunit ribosomal RNA gene, partial sequence	983	983	100%	100.00%	MK392015.1

Journal of *Fungi*

MDPI

Table S1 (continue)

		Fusarium redolens strain MLS02 small subunit ribosomal RNA gene, partial sequence; internal transcribed spacer 1, 5.8S ribosomal RNA gene, and internal transcribed spacer 2, complete sequence; and large subunit ribosomal RNA gene, partial sequence	983	983	100%	100.00%	OR523626.1
		Fusarium oxysporum strain CF10G small subunit ribosomal RNA gene, partial sequence; internal transcribed spacer 1, 5.8S ribosomal RNA gene, and internal transcribed spacer 2, complete sequence; and large subunit ribosomal RNA gene, partial sequence	983	983	100%	100.00%	OQ629145.1
**SBUG-M1748**	***Fusarium oxysporum***	*Fusarium oxysporum* isolate PS3_5_2 small subunit ribosomal RNA gene, partial sequence; internal transcribed spacer 1, 5.8S ribosomal RNA gene, and internal transcribed spacer 2, complete sequence; and large subunit ribosomal RNA gene, partial sequence	983	983	100%	100.00%	MN909341.1
		Fusarium oxysporum clone QT303(7) small subunit ribosomal RNA gene, partial sequence; internal transcribed spacer 1, 5.8S ribosomal RNA gene, and internal transcribed spacer 2, complete sequence; and large subunit ribosomal RNA gene, partial sequence	983	983	100%	100.00%	OL744590.1
		Fusarium oxysporum isolate GY28 small subunit ribosomal RNA gene, partial sequence; internal transcribed spacer 1, 5.8S ribosomal RNA gene, and internal transcribed spacer 2, complete sequence; and large subunit ribosomal RNA gene, partial sequence	981	981	99%	100.00%	OR789482.1
		Fusarium oxysporum isolate GY24 small subunit ribosomal RNA gene, partial sequence; internal transcribed spacer 1, 5.8S ribosomal RNA gene, and internal transcribed spacer 2, complete sequence; and large subunit ribosomal RNA gene, partial sequence	981	981	99%	100.00%	OR789478.1
		Fusarium oxysporum genomic DNA sequence contains ITS1, 5.8S rRNA gene, ITS2, isolate CIB_17	981	981	99%	100.00%	LN828193.1
		Fusarium oxysporum strain sj6-2 small subunit ribosomal RNA gene, partial sequence; internal transcribed spacer 1, 5.8S ribosomal RNA gene, and internal transcribed spacer 2, complete sequence; and large subunit ribosomal RNA gene, partial sequence	981	981	99%	100.00%	OM883861.1
		Fusarium oxysporum clone QT324(7) small subunit ribosomal RNA gene, partial sequence; internal transcribed spacer 1, 5.8S ribosomal RNA gene, and internal transcribed spacer 2, complete sequence; and large subunit ribosomal RNA gene, partial sequence	981	981	99%	100.00%	OL744599.1
**SBUG-M1768**	***Fusarium oxysporum***	*Fusarium oxysporum* f. sp. ciceris isolate FOCAMU5 internal transcribed spacer 1, partial sequence; 5.8S ribosomal RNA gene and internal transcribed spacer 2, complete sequence; and large subunit ribosomal RNA gene, partial sequence	824	824	99%	100%	PP217446.1
		Fusarium oxysporum isolate AK2 small subunit ribosomal RNA gene, partial sequence; internal transcribed spacer 1, 5.8S ribosomal RNA gene, and internal transcribed spacer 2, complete sequence; and large subunit ribosomal RNA gene, partial sequence	824	824	99%	100%	OR915474.1

Table S1 (continue)

		Fusarium foetens isolate P101D_F internal transcribed spacer 1, partial sequence; 5.8S ribosomal RNA gene and internal transcribed spacer 2, complete sequence; and large subunit ribosomal RNA gene, partial sequence	824	824	99%	100%	OR905647.1
		Fusarium foetens isolate M101D_F internal transcribed spacer 1, partial sequence; 5.8S ribosomal RNA gene and internal transcribed spacer 2, complete sequence; and large subunit ribosomal RNA gene, partial sequence	824	824	99%	100%	OR905634.1
		Fusarium foetens isolate M81B_F internal transcribed spacer 1, partial sequence; 5.8S ribosomal RNA gene and internal transcribed spacer 2, complete sequence; and large subunit ribosomal RNA gene, partial sequence	824	824	99%	100%	OR905620.1
		Fusarium oxysporum isolate F240 small subunit ribosomal RNA gene, partial sequence; internal transcribed spacer 1, 5.8S ribosomal RNA gene, and internal transcribed spacer 2, complete sequence; and large subunit ribosomal RNA gene, partial sequence	824	824	99%	100%	OR123360.1
		Fusarium oxysporum isolate F169 small subunit ribosomal RNA gene, partial sequence; internal transcribed spacer 1, 5.8S ribosomal RNA gene, and internal transcribed spacer 2, complete sequence; and large subunit ribosomal RNA gene, partial sequence	824	824	99%	100%	OR123329.1
		Fusarium oxysporum isolate F127 internal transcribed spacer 1, partial sequence; 5.8S ribosomal RNA gene and internal transcribed spacer 2, complete sequence; and large subunit ribosomal RNA gene, partial sequence	824	824	99%	100%	OR123312.1
		Fusarium oxysporum isolate F91 small subunit ribosomal RNA gene, partial sequence; internal transcribed spacer 1, 5.8S ribosomal RNA gene, and internal transcribed spacer 2, complete sequence; and large subunit ribosomal RNA gene, partial sequence	824	824	99%	100%	OR123300.1
		Fusarium oxysporum isolate F17 small subunit ribosomal RNA gene, partial sequence; internal transcribed spacer 1, 5.8S ribosomal RNA gene, and internal transcribed spacer 2, complete sequence; and large subunit ribosomal RNA gene, partial sequence	824	824	99%	100%	OR123279.1
**SBUG-M1769**	***Fusarium oxysporum***	*Fusarium oxysporum* f. sp. ciceris isolate FOCAMU5 internal transcribed spacer 1, partial sequence; 5.8S ribosomal RNA gene and internal transcribed spacer 2, complete sequence; and large subunit ribosomal RNA gene, partial sequence	821	821	100%	100%	PP217446.1
		Fusarium oxysporum isolate AK2 small subunit ribosomal RNA gene, partial sequence; internal transcribed spacer 1, 5.8S ribosomal RNA gene, and internal transcribed spacer 2, complete sequence; and large subunit ribosomal RNA gene, partial sequence	821	821	100%	100%	OR915474.1

Table S1 (continue)

		Fusarium foetens isolate P101D_F internal transcribed spacer 1, partial sequence; 5.8S ribosomal RNA gene and internal transcribed spacer 2, complete sequence; and large subunit ribosomal RNA gene, partial sequence	821	821	100%	100%	OR905647.1
		Fusarium foetens isolate M101D_F internal transcribed spacer 1, partial sequence; 5.8S ribosomal RNA gene and internal transcribed spacer 2, complete sequence; and large subunit ribosomal RNA gene, partial sequence	821	821	100%	100%	OR905634.1
		Fusarium foetens isolate M81B_F internal transcribed spacer 1, partial sequence; 5.8S ribosomal RNA gene and internal transcribed spacer 2, complete sequence; and large subunit ribosomal RNA gene, partial sequence	821	821	100%	100%	OR905620.1
		Fusarium oxysporum isolate F240 small subunit ribosomal RNA gene, partial sequence; internal transcribed spacer 1, 5.8S ribosomal RNA gene, and internal transcribed spacer 2, complete sequence; and large subunit ribosomal RNA gene, partial sequence	821	821	100%	100%	OR123360.1
		Fusarium oxysporum isolate F169 small subunit ribosomal RNA gene, partial sequence; internal transcribed spacer 1, 5.8S ribosomal RNA gene, and internal transcribed spacer 2, complete sequence; and large subunit ribosomal RNA gene, partial sequence	821	821	100%	100%	OR123329.1
		Fusarium oxysporum isolate F127 internal transcribed spacer 1, partial sequence; 5.8S ribosomal RNA gene and internal transcribed spacer 2, complete sequence; and large subunit ribosomal RNA gene, partial sequence	821	821	100%	100%	OR123312.1
		Fusarium oxysporum isolate F91 small subunit ribosomal RNA gene, partial sequence; internal transcribed spacer 1, 5.8S ribosomal RNA gene, and internal transcribed spacer 2, complete sequence; and large subunit ribosomal RNA gene, partial sequence	821	821	100%	100%	OR123300.1
		Fusarium oxysporum isolate F17 small subunit ribosomal RNA gene, partial sequence; internal transcribed spacer 1, 5.8S ribosomal RNA gene, and internal transcribed spacer 2, complete sequence; and large subunit ribosomal RNA gene, partial sequence	821	821	100%	100%	OR123279.1
**SBUG-M1770**	***Penicillium javanicum***	*Penicillium meloforme* clone SF_297 internal transcribed spacer 1, partial sequence; 5.8S ribosomal RNA gene and internal transcribed spacer 2, complete sequence; and large subunit ribosomal RNA gene, partial sequence	902	902	100%	100%	MT529573.1
		Penicillium meloforme clone EF_622 internal transcribed spacer 1, partial sequence; 5.8S ribosomal RNA gene and internal transcribed spacer 2, complete sequence; and large subunit ribosomal RNA gene, partial sequence	902	902	100%	100%	MT529271.1

Table S1 (continue)

Penicillium meloforme clone EF_621 internal transcribed spacer 1, partial sequence; 5.8S ribosomal RNA gene and internal transcribed spacer 2, complete sequence; and large subunit ribosomal RNA gene, partial sequence	902	902	100%	100%	MT529270.1
Penicillium meloforme clone EF_612 internal transcribed spacer 1, partial sequence; 5.8S ribosomal RNA gene and internal transcribed spacer 2, complete sequence; and large subunit ribosomal RNA gene, partial sequence	902	902	100%	100%	MT529261.1
Penicillium caperatum isolate CMV001H1 18S ribosomal RNA gene, partial sequence; internal transcribed spacer 1, 5.8S ribosomal RNA gene, and internal transcribed spacer 2, complete sequence; and 28S ribosomal RNA gene, partial sequence	902	902	100%	100%	MK450677.1
Penicillium caperatum isolate CMV013G2 18S ribosomal RNA gene, partial sequence; internal transcribed spacer 1, 5.8S ribosomal RNA gene, and internal transcribed spacer 2, complete sequence; and 28S ribosomal RNA gene, partial sequence	902	902	100%	100%	MK951937.1
Penicillium javanicum culture CBS:129395 strain CBS 129395 small subunit ribosomal RNA gene, partial sequence; internal transcribed spacer 1, 5.8S ribosomal RNA gene, and internal transcribed spacer 2, complete sequence; and large subunit ribosomal RNA gene, partial sequence	902	902	100%	100%	MH865296.1
Penicillium javanicum culture CBS:291.53 strain CBS 291.53 small subunit ribosomal RNA gene, partial sequence; internal transcribed spacer 1, 5.8S ribosomal RNA gene, and internal transcribed spacer 2, complete sequence; and large subunit ribosomal RNA gene, partial sequence	902	902	100%	100.00%	MH857207.1
Penicillium javanicum isolate Y9_ITS1F internal transcribed spacer 1, partial sequence; 5.8S ribosomal RNA gene and internal transcribed spacer 2, complete sequence; and large subunit ribosomal RNA gene, partial sequence	902	902	100%	100%	MF574327.1
Eupenicillium sp. LG41 genomic DNA sequence contains ITS1, 5.8S rRNA gene and ITS2, isolate LG41	902	902	100%	100%	LN626295.1

a) First ten hits of the NCBI (National Centre for Biotechnology Information; https://www.ncbi.nlm.nih.gov/) using nucleotide Basic Local Alignment Search Tool (BLASTn; accessed on 20. February 2024). **b)** Naming is based on the proximity of the query sequences to the majority of the given 100 hits using blast tree viewer tool.

Table S2. Macroscopic characterization of the filamentous fungi after 7 days of growth on MAg medium.

Strain	Color	Growth	Visual observation
***Aspergillus* sp. SBUG-M1743**	White	+++	Round uniform foci distributed over the entire plate interthread to form a dense mat of could-like cell mass. After 7 days the fungal biomass turned into dark spots
***Penicillium javanicum* SBUG-M1744**	Brown-beige	+++	Fungi grew in the form of small numerous foci with the appearance of individual larger dots. Gray spots started to appear at the corner of some foci after 7 days
***Trichoderma harzianum* SBUG-M1750**	Dark green	++	Growth started on the starting agar pieces in the form of round foci with numerous grainy cells distributed over the entire plate.
***Fusarium oxysporum* SBUG-M1746 and SBUG-M1748**	White/light beige	++	Cell biomass was in the form of threads that first appeared mainly on the starting agar pieces to make fur-like structure. With time, most of the medium was covered with round uniform foci. Fine and thin cloud-like cell mat makes demarcation on most of the plates.
***Fusarium oxysporum* SBUG-M 1768 and SBUG-M1769**	White to pinkish	++	Mycelia grew around the agar pieces, with some being aerial. With growth, pink mycelia gradually developing toward the out edges.
***Penicillium javanicum* SBUG-M1770**	White turned to greyish-green	++	White mycelial colony followed by the formation of separate threads that turned greyish-green..

++ good growth; +++ strong growth Inoculation. Inoculations were done via three-point method.

Table S3. Growth of SBUG-M1743, SBUG-M1744, SBUG-M1750, SBUG-M1768, SBUG-M1769 and SBUG-M1770 on the substrate tetradecane.

Strain	Inocluation technique	Growth 5 d	Growth 7 d	Visual observation
***Aspergillus* sp. SBUG-M1743**	Three-point method	+	++	Growth appeared as round foci around the starting agar pieces with white fine could-like growth that became clearly visible with strong demarcation after 7 days.
	Streak	++	+++	White fine could-like dots covered the entire plate.
***Penicillium ja-vanicum* SBUG-M1744**	Three-point method	++	++	Individual yellow dense mats appear after 7 days of growth. Clear boundaries and round foci around the starting agar pieces were also visible.
	Streak	++	+++	Yellowish-white growth was observed at the plate centre. The entire plate was covered with cloud-like dotted trails.
***Trichoderma harzianum* SBUG-M1750**	Three-point method	-	+	A few dark-green fine cells appeared near the starting agar pieces.
	Streak	-	+	The entire plate was covered with dark green cell traces.
***Fusarium oxysporum* SBUG-M1768 & SBUG-M1769**	Three-point method	++	++	Individual white dense mats around the starting agar pieces after 7 days
	Streak	++	++	White fine cloud-like dots covered the nearer and further surroundings of the streak after 7 days
***Penicillium ja-vanicum* SBUG-M1770**	Three-point method	++	+++	Same as SBUG-M1744
	Streak	++	+++	

-no growth; + weak growth; ++ good growth; +++ strong growth

Journal of Fungi

Table S4. Growth of SBUG-M1746, and SBUG-M1748 on their isolation substrate cyclohexanone.

Strain	Inocluation technique	Growth 5 d	Growth 7 d	Visual observation
Fusarium oxysporum **SBUG-M1746**	Three-point method	-	+	Crystal like white beige growth is characterized by jagged edges and round foci around the starting agar pieces
	Streak	-	++	White cotton-like growth moderately covered the entire plate.
Penicillium javanicum **SBUG-M 1748**	Three-point method	++	++	At the edges of the starting agar pieces clear boundaries of cloud white growth was formed.
	Streak	-	++	The entire plate was covered with cotton-like white growth

-no growth; +weak growth; ++good growth
